# Mechanisms of TGFβ-Induced Epithelial–Mesenchymal Transition

**DOI:** 10.3390/jcm5070063

**Published:** 2016-06-29

**Authors:** Aristidis Moustakas, Carl-Henrik Heldin

**Affiliations:** 1 Ludwig Cancer Research, Science for Life Laboratory, Uppsala University, Box 595, SE 751 24 Uppsala, Sweden; 2 Department of Medical Biochemistry and Microbiology, Science for Life Laboratory, Uppsala University, Box 582, SE 751 23 Uppsala, Sweden

**Keywords:** epithelial-mesenchymal transition, signal transduction, transcription factor, transforming growth factor β, tumor invasiveness

## Abstract

Transitory phenotypic changes such as the epithelial–mesenchymal transition (EMT) help embryonic cells to generate migratory descendants that populate new sites and establish the distinct tissues in the developing embryo. The mesenchymal descendants of diverse epithelia also participate in the wound healing response of adult tissues, and facilitate the progression of cancer. EMT can be induced by several extracellular cues in the microenvironment of a given epithelial tissue. One such cue, transforming growth factor β (TGFβ), prominently induces EMT via a group of specific transcription factors. The potency of TGFβ is partly based on its ability to perform two parallel molecular functions, i.e. to induce the expression of growth factors, cytokines and chemokines, which sequentially and in a complementary manner help to establish and maintain the EMT, and to mediate signaling crosstalk with other developmental signaling pathways, thus promoting changes in cell differentiation. The molecules that are activated by TGFβ signaling or act as cooperating partners of this pathway are impossible to exhaust within a single coherent and contemporary report. Here, we present selected examples to illustrate the key principles of the circuits that control EMT under the influence of TGFβ.

## 1. Introduction

### 1.1. EMT from a Signaling Pathway Perspective

**Figure 1:**
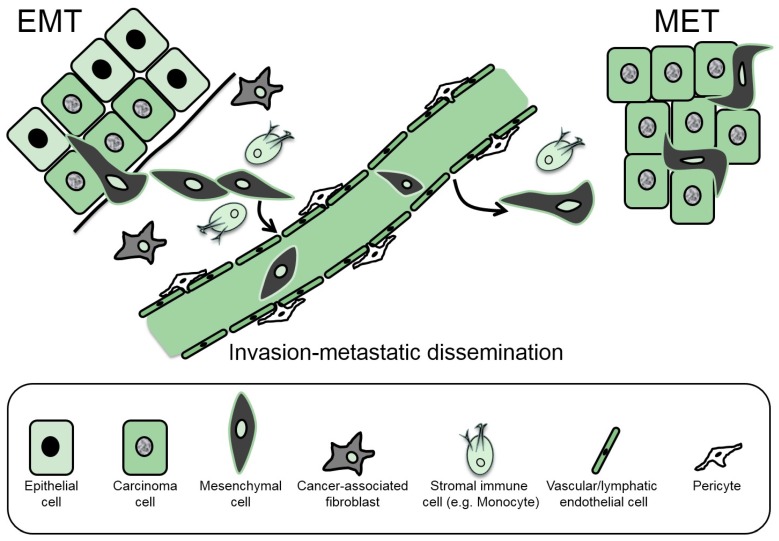
Schematic representation of EMT (epithelial–mesenchymal transition) and MET (mesenchymal–epithelial transition) in the context of carcinoma progression. Hyperplastic epithelial cells (light green cytoplasm) are shown aligned along their basement membrane (thick black line). Carcinoma cells develop in this primary tumor (deep green cytoplasm) and some undergo EMT, forming mesenchymal cells (black cytoplasm) that degrade the basement membrane and invade the local microenvironment. EMT and invasiveness are enhanced by auxiliary paracrine signals from stromal fibroblasts and immune cells, thus facilitating the movement of mesenchymal cells to the blood or lymphatic vessels where they can intravasate. Upon successful survival in the lymphatic or vascular circulation, some mesenchymal cells extravasate and initiate micrometastases consisting of mesenchymal cells and a bulk of carcinoma cells (deep green cytoplasm) that are generated via MET. The cell types of the schematic are explained below the picture.

The cells of a developing blastocyst have epithelial characteristics, and this underscores the importance of phenotypic transitions, often known as transdifferentiations, that bestow progenitor cells with the properties that are necessary for the generation of new tissues. Accordingly, the epithelial–mesenchymal transition (EMT) is a developmental transdifferentiation process (Figure 1) that defines many characteristic changes during early (e.g., primitive streak formation), intermediate (neural crest formation) and late (palatal roof closure and cardiac septal formation) embryonic development [1]. These developmental EMTs are frequently coupled with the inverse process, i.e., mesenchymal–epithelial transition (MET) (Figure 1), which ensures the generation of new epithelia from the migratory mesenchymal progenitor cells after their relocation to a new embryonic site [1]. In addition, a broad definition of epithelial tissues would also include the barrier-generating cells of the blood and lymphatic vessel walls, thus explaining why EMT is also observed in vascular endothelial and lymphatic endothelial cells, often referred to as EndoMT [2,3]. Such developmental EMTs have been termed type I EMTs to distinguish them from the EMTs seen in pathological conditions, such as the type II EMTs that operate during wound healing and tissue fibrosis, and the type III EMTs that participate during the progression of cancer [4].

**Figure 2:**
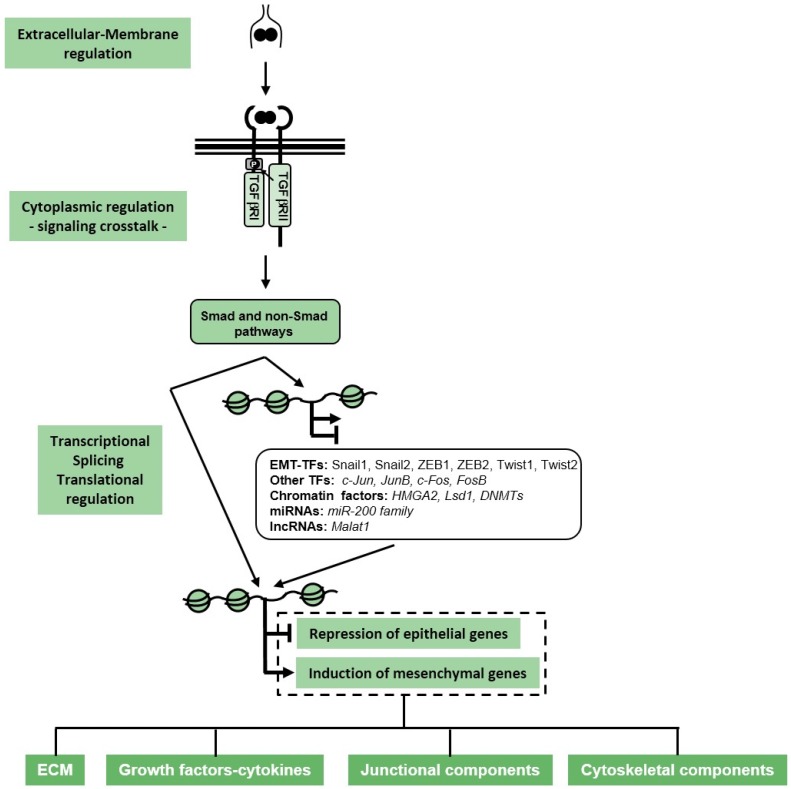
Summary of TGFβ signaling during EMT. Inactive TGFβ ligand (bound to its *N*-terminal latency associated peptide) is present in the extracellular space. After activation, TGFβ binds to the cell surface receptors. In the ligand-bound receptor complex, the type II receptor (TGFβRII) phosphorylates the juxtamembrane domain (small box with the symbol P) of the type I receptor (TGFβRI), which causes activation of Smad or other signaling proteins (collectively referred to as non-Smad pathways). These pathways positively or negatively regulate gene transcription; examples of target genes are given in the figure. The products of these genes often cooperate with the Smad and non-Smad signaling pathways to regulate expression of a second tier of genes, leading to the downregulation of epithelial genes and the upregulation of mesenchymal genes. These genes are classified in four functional groups based on their primary cellular functions (bottom grey boxes). On the left side of the pathway, the regulatory mechanisms are highlighted.

**Figure 3:**
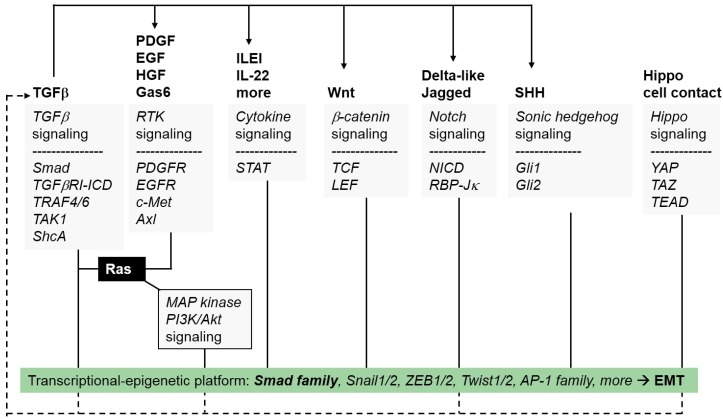
Crosstalk between TGFβ and other signaling pathways during EMT. TGFβ signaling is listed together with six additional signaling pathways. Each pathway is summarized above the dotted line. Secreted growth factors that initiate the pathways are listed in bold on the top. Signaling mediators are listed below the dotted line in the grey boxes. All pathways interact with each other in regulating the expression and/or activity of transcription factors that elicit the EMT. Examples of such transcriptional regulators are shown. The Ras small GTPase is highlighted due to its central role in the process of tumorigenesis and EMT. The downstream kinase pathways initiated by Ras are shown separately. TGFβ is known to induce expression of ligands for many other pathways (top arrows). Upon activation of transcription, many pathways induce the expression of TGFβ family ligands (bottom straight and dotted arrows).

When viewed from a molecular signaling perspective, the various EMTs, whether taking place during embryogenesis or during pathogenesis such as in cancer, seem to be initiated by physiological or abnormal activation of the major developmental signaling pathways, including the Wnt, Notch, receptor tyrosine kinase (RTK), cytokine receptor-JAK–signal transducer and activator of transcription (STAT), hedgehog, hippo, nuclear receptor and transforming growth factor β (TGFβ) pathways (Figure 2 and Figure 3) [5,6]. The activities of these pathways appear to be interconnected with each other and, depending on the specific epithelial or endothelial cell type affected, different signaling mediators or controlling molecules mediate their interconnection or crosstalk (Figure 3). As discussed later for TGFβ, an important phenomenon operating at least during cancer EMTs is the ability of a single cytokine to promote the secretion of multiple other growth factors, thus enhancing the transdifferentiation process (Figure 2 and Figure 3) [7,8]. In addition, the activities of the various developmental signaling pathways seem to orchestrate a conserved and redundant cellular response that involves at least three programs (Figure 2): (a) The epithelial cells responding to the pro-EMT growth factors induce the neosynthesis of many extracellular matrix (ECM) and cell surface proteins, causing an architectural remodeling of the local environment at the surface of the transitioning cell. (b) The coordinate action of growth factors and remodeled ECM enforce an alteration of most of the intercellular adhesion complexes, leading to the generation of new functional complexes that mediate cellular plasticity and allow for partial or even complete detachment of cells from their neighbors. These changes, together with the synthesis of integrins and ECM components, enhance the ability of the transitioned cells to migrate. (c) A corresponding remodeling of the cytoskeleton takes place and further supports the new motility requirements of the transitory mesenchymal cells. All these cell biological modifications occur via molecular events that act at two distinct subcellular levels: (a) At the plasma membrane and cytoplasmic level, signaling molecules activate processes of receptor endocytosis, protein ubiquitylation and lysosomal degradation, altogether mediating the loss of epithelial multi-protein assemblies and orchestrating the cytoskeletal remodeling that supports cell motility. (b) At the nuclear level, newly synthesized transcription factors (EMT transcription factors, EMT-TFs) and splicing factors enforce epigenetic changes, thus reprogramming the repertoire of gene expression that mediates the loss of epithelial properties and the generation of the mesenchymal, transitory cell phenotype. The best understood EMT-TFs belong to the Snail family of zinc finger proteins (Snail1, Snail2), the ZEB family of zinc finger and homeodomain proteins (ZEB1, ZEB2) and the Twist family of basic helix loop helix proteins (Twist1, Twist2, Twist3) [9]. The nuclear and the cytoplasmic activities are tightly interconnected such that cytoplasmic events are required for the activation of the nuclear gene expression program, and gene activity is necessary to provide key molecules that drive the mesenchymal changes in the cytoplasm or at the plasma membrane.

### 1.2. Basic Aspects of TGFβ Signal Transduction

Although many extracellular growth factors mediate EMTs, this article focuses on TGFβ, which is the prototype of a family of 33 or more secreted developmental factors in humans, including three distinct gene products, TGFβ1, TGFβ2 and TGFβ3, as well as the bone morphogenetic proteins (BMPs), activins and the Müllerian inhibiting substance (MIS) [10]. TGFβ and most of its family members are secreted as latent, inactive, disulfide-linked polypeptide dimers; TGFβ isoforms are deposited in the ECM of every tissue and into the granules of platelets [11]. Latent TGFβ is activated by the proteolytic cleavage of several ECM-associated polypeptides that tether the latent ligand to the ECM, or by integrin receptor-mediated conformational changes to the latent precursor so that the bioactive *C*-terminal dimeric polypeptide is liberated and presented to the signaling receptors [12].

The bioactive ligand binds to its cell surface receptors, known as the type II and type I receptors of the TGFβ family, which have intrinsic serine/threonine and weaker tyrosine kinase activity [6]. Ligand-bound type II receptor trans-phosphorylates the type I receptor within a hetero-tetrameric receptor complex, causing type I receptor kinase activation. The hetero-tetrameric ligand–receptor complex recruits adaptors and activates ubiquitin ligases of the tumor necrosis factor α receptor associated factor (TRAF) family. This leads to the activation of mitogen activated protein (MAP) kinase pathways and cleavage of the type I receptor intracellular domain which moves to the nucleus and regulates the expression of genes that control the EMT [13,14]. The type I receptor also phosphorylates the *C*-terminal tail of receptor-activated (R-) Smad proteins, such as Smad2 and Smad3, which form complexes with Smad4 in the nucleus that bind directly or indirectly to DNA, thus providing additional regulation of target genes that mediate the EMT and many other cellular responses [7].

Inhibitory Smads, Smad6 and Smad7, are induced by incoming TGFβ signaling inputs and provide negative control on the type I receptors and Smads. The inhibitory Smads block the flow of TGFβ signaling either by enhancing receptor ubiquitylation and lysosomal degradation or by inactivating R-Smad/Smad4 complexes [10,11]. Inhibitory Smads also participate in the activation of the TRAF family ubiquitin ligases and MAP kinases that are stimulated by the TGFβ receptor complex [13,15]. Altogether, these signaling mediators provide a complex network of positive and negative regulatory steps that ensure limited and quantitative action of TGFβ in diverse tissue types, while many of these mechanisms are misregulated in cancer cells [16]. As analyzed further below, all these signaling mechanisms are implicated in the control of the EMT (Figure 2).

## 2. The Cellular Basis of EMT

At the cellular level, the manifestation of EMT is usually described as including two parallel programs; first, the destruction (partial or complete) of epithelial differentiation properties and second, the generation of the mesenchymal program (Figure 1). Research activity has focused more intensely on the gradual loss of epithelial differentiation and less on the acquisition of the mesenchymal program. Hallmarks of these two cell biological changes include the replacement of E-cadherin by N-cadherin in the adherens junctions of cells that undergo EMT, the expression of new cytoskeletal filaments such as the intermediate filaments containing vimentin or specific cytokeratins and the profound induction of proteins and glycosaminoglycans of the ECM that link to the enhanced invasiveness of the cells generated via the EMT [9]. In the following sections, we present an overview of these two differentiation programs by selecting examples that link them to the actions of TGFβ as a central inducer of the EMT.

### 2.1. ECM Changes Associated with EMT

The extensive remodeling of the ECM that takes place during EMT provides molecular support for the completion of the transition but also appears as an end-product of the process. This feature of EMT is especially relevant to the fibrotic aspects of either chronic inflammatory disorders or cancer, the later in which the tumor stroma appears progressively desmoplastic as the disease advances. TGFβ is known to exhibit strong profibrotic signals and this is one of its major functions in adult tissues and during the process of wound healing [12]. It has been proposed that key ECM components and other mesenchymal-associated genes are co-regulated and transcriptionally induced by the forkhead transcription factor FoxC2 [17]. It will be of interest to analyze the involvement of FoxC2 during ECM synthesis under the influence of TGFβ. The best characterized ECM components that are induced by TGFβ signaling include specific collagen family members, which exhibit cell type specificity, and fibronectin, which is induced in a rather universal manner. TGFβ also induces the synthesis of secreted regulators and surface receptors that signal via ECM protein associations, including the matrix metalloproteases (MMP2, MMP9 and more), integrin receptors and plasminogen activator inhibitor 1 (PAI-1) [18,19]. The genes for these important ECM components are induced via Smads, Jun *N*-terminal kinase (JNK) and p38 MAP-kinase [18,20] and TGFβ-activated kinase 1 (TAK1), which regulates nuclear factor κ B (NF-κB) transcriptional activity during TGFβ-induced MMP9 synthesis [21]. MMP activity is important for cell migration and penetration through the basement membrane of epithelial organs during the healing of wounded epithelia or carcinoma cell invasiveness. Such invasion elicited after the induction of EMT is transcriptionally controlled by the action of Twist1, a major EMT-TF that promotes the expression of platelet-derived growth factor (PDGF) α-receptor and many integral membrane proteins that mediate the formation of invadopodia, key functional assemblies of the plasma membrane of invading mesenchymal cells [22].

Similar to the point we will discuss later about the reciprocal roles of TGFβ and other cytokines, ECM components such as fibronectin and MMPs are not only induced by TGFβ signaling in mammary epithelial cells, but can themselves provide critical signals that promote the EMT, including control of the activation of TGFβ ligand in the extracellular environment [23]. The membrane type I-matrix metalloprotease (MT1-MMP or MMP14) promotes EMT and invasiveness by activating extracellular TGFβ, which then induces the secretion of Wnt5a and thus provides a paracrine signal to epithelial prostate cancer cells [24]. A similar action can be observed for the extracellular glycosaminoglycan hyaluronan, whose synthesis can be induced by TGFβ signaling; hyaluronan activates TGFβ signaling and its downstream transcriptional mediators Snail1 and Twist1, thus promoting EMT and the enrichment of tumor cells in cancer stem cell populations [25]. The evidence for this concept was demonstrated in a transgenic mouse model that expresses one of the potent biosynthetic enzymes of hyaluronan, hyaluronan synthase 2 (HAS2) [25]. Interestingly, TGFβ-induced EMT in mammary epithelial cells depends on the transcriptional induction of HAS2, however, removal of extracellular hyaluronan using recombinant hyaluronidases or by blocking the major hyaluronan signaling receptor, CD44, could not inhibit the HAS2-induced EMT, suggesting there is a possible hyaluronan-independent role for HAS2 in the process of EMT [26]. Thus, as EMT progresses, TGFβ induces fibronectin, hyaluronan and other ECM molecules that in turn sensitize those cells to respond to TGFβ. This is corroborated by experiments done using an artificial ECM where the stiffness of the matrix could be controlled; when the matrix was soft, TGFβ promoted apoptosis, whereas TGFβ induced EMT when the matrix was made rigid [27]. One possible mechanism by which ECM stiffness may regulate TGFβ signaling involves crosstalk between the Hippo pathway and its transcriptional mediators, YAP/TAZ, which directly interact with Smad proteins and modulate their residence time within the nucleus (Figure 3) [28].

TGFβ signaling promotes a global switch in the content of specific *N*-linked glycans that decorate ECM and cell surface proteins [29]. Among the glycosylation changes that occur during TGFβ-induced EMT, modification of proteins based on sialic acid moieties appears to play important functional roles, as blocking the enzymes that catalyze protein sialylation enhances the EMT response [30]. While protein sialylation may affect a large number of cell surface or secreted proteins, one specific case is especially important to EMT. Fucosylation of the TGFβ type I receptor, which is mediated by fucosyltransferase 3 and 6 in colorectal cancer cells, is required for normal signaling activity of the receptor, induction of the EMT and tumor cell invasiveness [31]. The ECM, with its multifunctional constituents, provides a fertile source of TGFβ (see below) and other growth factors that guide the EMT process.

### 2.2. EMT Involves Changes in Secreted Growth Factors and Cytokines

Similar to the interplay between TGFβ and ECM components, multiple cytokines are activated in a sequential manner during the onset and progression of the EMT program (Figure 3). This mode of action is thought to offer a robust and interdependent network of synergizing growth factors [32]; however, the necessity of such a network varies from one epithelial tissue to another. In hepatocellular carcinoma (HCC) cells, EMT stimulated by TGFβ activates both Wnt and sonic hedgehog (Shh) signaling and this is required for the establishment of a stable EMT (Figure 3). These pathways operate via alternative feedback mechanisms that were shown to be important based on mathematical modeling of the behavior of distinct epithelial and mesenchymal HCC cell models [33]. Strong evidence also links the action of TGFβ with the secretion of epidermal growth factor (EGF), PDGF and cytokines of the extended interleukin family during the course of EMT (Figure 3). In breast cancer cells, EGF secretion stimulated by TGFβ promotes EMT and cancer cell invasion by activation of focal adhesion kinase, the activity of which is required for the proper coupling of integrin receptors to the cytoskeletal apparatus during cell migration [34]. EGF receptor signaling is also modulated during EMT induced by TGFβ; TGFβ downregulates micro-RNA 200 (miR-200) expression, which normally acts as a negative regulator of the mitogen-inducible gene 6 (MIG6) gene. Upon TGFβ stimulation, MIG6 is released from the negative regulation by miR-200 and enforces negative control on the EGF receptor [35]. The derepression of MIG6 by TGFβ allows the EGF receptor to signal through the downstream Akt kinase in a constitutive manner, thus partially explaining how cells that undergo EMT develop resistance to EGF receptor inhibitors, such as erlotinib. Such mechanisms help lung or pancreatic cancer cells to progress towards more malignant stages [35]. In addition to EGF, lung adenocarcinoma cells undergoing EMT secrete Shh (Figure 3), and blocking the activity of this pathway suppresses the TGFβ-induced EMT in these cells [36]. Alternatively, lung fibrosis in asthmatic patients depends on TGFβ, which promotes secretion of interleukin 22 (IL-22), synergizing with TGFβ to promote EMT in bronchial epithelial cells (Figure 3) [37].

The chemokine CCL21 is induced by TGFβ in lymphatic endothelial cells and acts on invading breast cancer cells that undergo EMT, thus permitting a functional crosstalk between the tumor cell and the lymphatic system [38]. In this manner, TGFβ appears capable of sensitizing both the tumor cell and the lymphatic endothelium to CCL21, so that it can signal and promote the chemotactic migration of the tumor cells towards the lymphatic vessels. Chemotactic migration and regulation of tumor vasculature is a strong feature of PDGF signaling, and the specific crosstalk of this pathway with TGFβ is best demonstrated in liver cancer models. A transgenic mouse model of HCC with oncogenic K-Ras expression in the liver exhibits strong synergistic cooperation between Ras–MAP kinase and TGFβ signaling, promoting EMT and liver cancer metastasis [39]. TGFβ induces the secretion of interleukin-like EMT-inducer (ILEI) which promotes EMT and metastasis (Figure 3) [39,40]. In this mechanism, ILEI then leads to the induction of PDGF receptor expression in the hepatocarcinoma cells, which further signals via STAT3 and β-catenin to establish a stable mesenchymal phenotype. Thus, ILEI and PDGF synergize during the induction of HCC EMT [40]; pharmacological administration of combined PDGF receptor and TGFβ receptor inhibitors can be effective at reverting EMT and reducing metastatic dissemination [41]. Whether the ILEI–PDGF signaling connection also mediates angiogenic growth in HCC remains to be examined. These selected studies enforce the paradigm that effective anti-metastatic therapy depends on cocktails of inhibitors that block two or possibly more interconnected signaling pathways stimulated by different growth factors.

### 2.3. EMT Changes the Cell Junctional Complexes

The remodeling of cell junctional complexes is often referred to as a hallmark of EMT, and the best example of this is the downregulation of E-cadherin and the loss of E-cadherin-based adherens junctions [42]. TGFβ-mediated E-cadherin loss from mammary epithelial cells is not only required for EMT, but is also necessary for efficient colonization of breast cancer cells to the lung [43]. The junctional remodeling involves a change in cellular polarity complexes and the subsequent loss of tight junctions and other plasma membrane properties that define the function of normal epithelia [44]. Mesenchymal cells build new types of membrane junctions, including N-cadherin-based adherens junctions and integrin-ECM focal adhesion complexes that are usually linked to the enhanced migratory capacity of the mesenchymal cells [42]. The E-cadherin to N-cadherin switch during EMT is well established [42], but additional cadherins are also regulated by TGFβ in epithelial cells. Pulmonary fibrosis involves both excessive TGFβ signaling and EMT, and lung tissue fibrosis correlates with cadherin-11 (also known as osteoblastic, OB-cadherin) expression [45]. TGFβ induces cadherin-11 expression in lung adenocarcinoma cells and this upregulation is required for the EMT response, while on the other hand, mice with a homozygous loss of cadherin-11 present with a relative resistance to pulmonary fibrosis and reduced levels of TGFβ in the lung [45]. Furthermore, an antibody that neutralizes the homotypic interactions between cadherin-11 proteins proved beneficial in reducing pulmonary fibrosis in normal mice. In a parallel mechanism, thyroid epithelial cells undergoing EMT in response to TGFβ induce expression of cadherin-6 (also known as K-cadherin) and this cadherin is found expressed at high levels in aggressive thyroid carcinomas, thus classifying cadherin-6 as a TGFβ-inducible mesenchymal cadherin [46].

Regulation of epithelial junctional assemblies does not only involve the core architectural proteins of these junctions, but also involves accessory, regulatory components, including lipids of the plasma membrane. A sphingolipid switch promoted by TGFβ signaling based on the transcriptional repression of the synthetic enzyme UDP-Gal:β1-3galactosyl-transferase-4, which glycosylates gangliotetra-acylceramide, is of critical importance for the disassembly of the adherens junctions. This sphingolipid supports adherens junctions by forming so-called glycosynaptic membrane domains, which are depleted upon the loss of galactosyl-transferase and thus facilitate the disassembly of the junctions [47]. Furthermore, upstream regulators of junctional assembly are members of the polarity complexes that associate with the cytoplasmic face of tight and other junctional complexes [44]. TGFβ induces Snail1 expression, which represses the Crumbs3 gene, whose product is a key regulatory subunit of the apico-basal polarity complex; in this manner, cell polarity is altered during EMT and causes the subsequent disassembly of tight junctions [48]. TGFβ type II receptor signaling is itself directly linked to changes in the polarity complex. The type II receptor phosphorylates the polarity subunit Par6, leading to recruitment of the Smad ubiquitylation regulatory factor 1 (Smurf1) and the subsequent ubiquitylation and proteasomal degradation of the associated small GTPase RhoA, whose activity is required for tight junction assembly by inducing actin polymerization [44]. This mechanism also depends on a transcriptional signal provided by TGFβ/Smad to the staphylococcal nuclease and tudor domain containing 1 (SND1) gene, leading to synthesis of the SND1 transcriptional co-activator, which induces expression of Smurf1 prior to its targeting of the RhoA GTPase during EMT [49]. RhoA can also be negatively regulated by miR-155, which stalls RhoA mRNA translation [50], whereas TGFβ, by inducing miR-491-5p expression, downregulates the polarity subunit Par3 (a partner of Par6) [51], demonstrating that TGFβ signaling negatively regulates at least two key components of epithelial polarity during the onset of EMT.

Similar to the N-cadherin and cadherin-6 switch in mesenchymal cells, assembly of new focal adhesions is important for mesenchymal cell migration. The adaptor protein Hic-5 participates in the assembly of focal adhesions and facilitates signaling by the small GTPase RhoC, which leads to MMP activation and formation of invadopodia by migratory mesenchymal cells [52]. TGFβ signaling induces both Hic-5 expression and tyrosine phosphorylation of Hic-5 by the Src kinase, which is activated by TGFβ during EMT. Whether the Hic-5 gene is a direct target of Twist transcriptional activity downstream of TGFβ remains to be clarified. In addition, several integrin receptors are newly synthesized in mesenchymal cells while others are downregulated from the epithelial basal membrane during EMT. An example from breast cancer cells illustrates the co-dependency of EMT and breast cancer cell metastasis on two integrins, integrin-β1 and integrin-β3 [53]. Experimental silencing of integrin-β1 partially blocked the ability of TGFβ to induce EMT and cancer cell invasiveness [53]. However, integrin-β3 upregulation seemed to compensate for the loss of integrin-β1, demonstrating the dependence of mesenchymal cells on these integrin-mediated adhesion complexes. TGFβ signaling regulates multiple members of the integrin family securing a safe transition to the mesenchymal and pro-invasive phenotype.

Thus, a switch in diverse junctional components of epithelial cells during EMT appears as a necessary phenotypic alteration that is both a final aim of pro-EMT signaling and a progressive mediator of the transition.

### 2.4. EMT Changes the Acto-Myosin Machinery

The necessary adaptation of plasma membrane junctional and adhesion complexes during EMT is associated with an intracellular reorganization of the cytoskeleton, including changes to the microfilaments, intermediate filaments and microtubules. This reorganization provides new capacities to mesenchymal cells in terms of the control of their proliferation, intercellular adhesion and ECM-based motility.

Cytoskeletal regulation controlled by small GTPases is an established theme during EMT [42]. The Rho GTPases require regulatory input from upstream guanine exchange factors, such as the Net1 enzyme, whose two isoforms, a nuclear and a cytoplasmic, are controlled by TGFβ signaling during EMT via direct transcriptional mechanisms as well as post-transcriptional regulation by miRNAs [54]. Interestingly, transcriptional induction of the cytoplasmic Net1 isoform takes place relatively fast, whereas after prolonged TGFβ signaling, the nuclear Net1 isoform is induced and cytoplasmic Net1 is downregulated because TGFβ induces miR-24, which targets Net1 [54]. The exact mechanism of activating cytoplasmic or nuclear Rho GTPase activity by regulating the Net1 GEF remains to be elucidated. During EMT, not only positive, but also negative regulation of Rho GTPase signaling takes place, as TGFβ signaling causes proteasomal degradation of two distinct GEFs, LARG and GEF-H1, resulting in decreased stiffness and remodeling of the cytoskeletal response of mesenchymal cells to integrin signals, so that invasiveness is enhanced [55]. Similarly, the adaptor protein lipoma preferred partner (LPP), a mesenchymal cell protein, is stabilized and translocates to focal adhesions in response to TGFβ signaling, promoting breast cancer invasiveness after the EMT [56]. LPP associates with α-actinin and the complex stabilizes actin microfilaments, thus positively contributing to cell migration.

Additional actin-binding proteins that operate at focal adhesions during mesenchymal cell migration include the adaptor proteins moesin [57] and zyxin, the latter of which switches its localization during EMT and promotes actin microfilament stability; TGFβ signaling induces zyxin mRNA levels with the help of Twist1 as a transcriptional regulator [58]. In lung cancer cells exhibiting enhanced invasiveness due to the TGFβ-induced EMT, the transcriptional induction of zyxin leads to a balanced expression of integrin receptors, causing proper focal adhesion assembly and ECM-based migration [59]. Regulation of actin microfilament and focal adhesion assemblies is critical during EMT and the ensuing cell migration, because cells contract and mobilize their plasma membrane based on myosin sliding on the actin fibers. The myosin IIB isoform is synthesized via alternative splicing induced by TGFβ signaling and correlates with the motility of breast epithelial cells that exhibit the EMT [60]. Similarly, melanoma cells that present a notoriously strong ability to invade and metastasize also exhibit TGFβ-induced EMT. In this model, the TGFβ receptors activate Smad2, which, together with one of its nuclear co-factors, Cbp/p300-interacting transactivator 1 (CITED1), regulates a large number of genes related to the acto-myosin machinery, including specific myosin light chain isoforms. This network may explain the characteristic ameboid motility that is exhibited by melanoma cells [61].

Myofibroblast differentiation is induced by TGFβ acting on fibroblasts or initially on epithelial cells that undergo epithelial-myofibroblast transition (EMyoT), a differentiation change highlighted by the expression of the specialized α-smooth muscle actin (αSMA) protein and its incorporation into contractile acto-myosin fibers [62]. Complex signaling interplay between TGFβ and fibroblast growth factor (FGF) is important during EMyoT. TGFβ induces the EMT transcription factors ZEB1 and ZEB2, which transcriptionally repress the epithelial splicing regulatory protein (ESRP) [63], blocking mammary epithelial splicing regulation and promoting alternative splicing of the FGF receptor 1, 2 and 3 IIIc isoforms. The alternative splicing of the FGF receptors renders the mesenchymal cells responsive to FGF2 and FGF4, which pushes them to the terminal myofibroblast stage [64]. Furthermore, TGFβ signaling leads to Snail2 expression followed by E-cadherin downregulation and the release of β-catenin from the adherens junctions. β-catenin promotes a transcriptional complex between the transcription factors myocardin-related transcription factor (MRTF) and serum-response factor (SRF), which cooperate to induce transcription of the αSMA gene in myofibroblasts generated via EMyoT [65,66,67]. Myofibroblast differentiation induced by TGFβ also involves multiple actin-regulatory proteins, including members of the Lin11, Isl-1 and Mec-3 (LIM) domain family and signaling kinases of the LIM kinase family (reviewed in [68]). It will be of interest to understand not only how EMT and EMyoT are linked to each other, but also how the same molecules mediate EMT under certain biological conditions while promoting EMyoT in a different context.

## 3. Regulatory Mechanisms that Control TGFβ-Induced EMT

### 3.1. Extracellular and Plasma Membrane Regulators

The epithelial cells undergoing EMT may secrete TGFβ in an autocrine manner, but more frequently, other cell types such as fibroblasts or immune cells provide TGFβ in a paracrine manner. The mechanisms that present or activate TGFβ in the tissue microenvironment are of importance for the EMT response and define the effectiveness of the paracrine action of this cytokine (Figure 2). An established scenario involves tumor cells that secrete chemokines, such as CXCL12, which act on cancer-associated fibroblasts (CAFs) and induce them to secrete TGFβ. The CAF-derived TGFβ then acts upon epithelial (carcinoma) cells and elicits the EMT; pharmacological inhibition of the secreted TGFβ, e.g., via a neutralizing antibody, has been shown to be effective at blocking the pro-EMT effects of breast cancer patient-derived CAFs on breast carcinoma cells [69]. Latent TGFβ that resides in the ECM of breast tumor cells can be activated by radiation treatment of patients, and this may generate signals that promote EMT in the malignant mammary epithelium [70]. TGFβ-induced EMT mediated by inflammatory cells in the tumor microenvironment is promoted by the leukotriene B4 receptor 2, which, in response to leukotriene B4, activates reactive oxygen species (ROS) and NF-κB transcriptional activity that facilitate the establishment of EMT by TGFβ [71]. Pharmacological inhibition or genetic interference with the leukotriene receptor abrogates the ability of TGFβ to induce mammary EMT. In a similar mode, bone marrow-derived mesenchymal stem cells (MSCs) are activated and express TGFβ that is bound to their cell surface-associated matrix, which then acts in a paracrine manner on colorectal cancer cells and causes the EMT [72]. For the MSC to deliver their TGFβ to the responding colorectal epithelial cells, direct cell–cell contact was required. The secreted small proteglycan biglycan is known to bind to extracellular TGFβ1 and modulates its access to the TGFβ receptors, a mechanism which can be regulated by the epithelial transcription factor p73 [73]. Loss of p73 in pancreatic tumor cells induces biglycan expression and secretion, leading to TGFβ activation and stimulation of EMT. Platelets also make direct contacts with breast cancer cells and secrete TGFβ, which mediates EMT and primes the tumor cells for metastases [74]. Accordingly, a mouse model with megakaryocyte/platelet-specific knock-out of the TGFβ1 gene exhibited less EMT and suppressed invasiveness [74]. Platelet–tumor cell contact activates NF-κB signaling in addition to the TGFβ response in the cancer cells, both contributing to the EMT and explaining how platelets promote metastasis.

Moreover, metastatic cells that exhibit signaling via TGFβ receptors, and the RTK Axl, secrete thrombospondin 2 and activate fibroblasts, which then provide a metastatic niche for distant organ colonization (e.g. breast cancer metastasis to lung) [75]. The ability of the pro-EMT tyrosine kinase receptor Axl to activate signaling by extracellular TGFβ is widespread and has also been documented in liver cancer cases of EMT [76]. According to this mechanism, Axl associates with the adaptor protein 14-3-3ζ and enhances phosphorylation of serine 213 in the linker domain of Smad3, switching TGFβ–Smad3 signaling towards a pro-EMT and pro-tumorigenic pathway that induces expression of Snail1 and pro-invasive MMP9 [76]. In addition, breast cancer cells that overexpress the EGF family receptor HER2 exhibit increased metastatic potential compared to mammary counterparts without HER2 overexpression, and this is partially due to the augmented secretion of autocrine TGFβ by such tumor cells, which enhances EMT and their pro-metastatic potential [77]. During lung fibrosis, pneumonocytes and adjacent fibroblasts enriched in the fibrotic wound express the chemokine CXCL9 and its receptor CXCR3; CXCL9 signaling activates Smad7 and suppresses TGFβ signaling in lung epithelial cells, thus providing a mechanism that counterbalances the pro-EMT and pro-fibrotic action of TGFβ in the lung [78].

Extracellular supply or activation of TGFβ is one aspect by which the tissue microenvironment acts on resident epithelial cells during cancer progression or fibrosis. Another aspect is the availability and regulation of receptors and signaling mediators on the target epithelial cells. One such negative regulator of EMT is the secreted protein fibulin-3, which binds to both TGFβ receptor kinases on the surface of breast cancer cells and limits their responsiveness to TGFβ. Thus, breast cancers with high fibulin-3 expression appear more epithelial and less aggressive, whereas breast tumors with low fibulin-3 expression exhibit mesenchymal features and enhanced invasiveness [79]. A related mechanism involves the kidney plasma membrane receptor, Klotho, whose extracellular domain can be shed after proteolytic cleavage. Klotho binds to the TGFβ receptor and blocks TGFβ signaling, thus limiting the EMT response, and protecting kidney cells from the pro-fibrotic action of TGFβ [80]. Klotho shedding can also regulate other receptor systems, including Wnt and insulin-like growth factor receptors [80]. The secreted protein signal peptide-CUB-EGF-like domain-containing protein 3 (SCUBE3) binds to TGFβRII and promotes TGFβ signaling via the same receptor, thus enhancing the EMT response in lung cancer cells [81]. In contrast, the related protein SCUBE2 seems to promote epithelial differentiation and block EMT; TGFβ signaling represses expression of this gene by enforcing methylation on the SCUBE2 DNA locus [82]. Whether SCUBE2 can also modulate TGFβ receptor signaling via direct binding to the receptors remains unexplored. TGFβRII availability can also be limited as a result of transcriptional repression of its gene due to activation of the histone deacetylase 1 (HDAC1) enzyme by the signaling protein CCN5 [83]. In an opposite scenario, the pro-metastatic homeobox transcription factor Six1 transactivates the gene encoding TGFβRI and promotes EMT by TGFβ [84].

TGFβ receptor sorting to the proper cell membrane compartments of epithelial cells can also impact on EMT. For instance, the TGFβ co-receptor betaglycan is normally expressed in the basolateral face of polarized breast epithelial cells; however, a betaglycan mutant that shows defective sorting results in a uniform distribution of the co-receptor and enhanced EMT [85]. Thus, a physiological co-receptor of TGFβ can modulate the signaling response and the final biological outcome towards EMT depending on the subcellular distribution of the receptor. Furthermore, lung EMT involves crosstalk between integrin receptor α3β1 and the TGFβ receptor, leading to the nuclear accumulation of Smad2 in complex with phosphorylated β-catenin, one mechanism which drives EMT in cultured A549 cells and in vivo during bleomycin-induced fibrosis [86]. The α3β1 integrin can also be differentially glycosylated by the *N*-acetyl-glucosaminyl-transferase III, whose levels are downregulated during TGFβ-induced EMT; overexpression of this enzyme protects the epithelial phenotype at least in part due to glycosylation and stabilization of E-cadherin levels on the plasma membrane [87].

The above examples suggest that many aspects of EMT that are controlled by extracellular or plasma membrane regulators of TGFβ remain to be understood and will provide new clues about potential multifactorial therapies against EMT in fibrosis or cancer.

### 3.2. Cytoplasmic Protein Regulators

TGFβ induces EMT in a variety of epithelial cell types by engaging almost all of the signaling modules that have been characterized downstream of its kinase receptors. These modules are often classified as Smad and non-Smad pathways, but as we illustrate here, the list of signaling mediators is large and includes both non-coding RNAs and proteins, including adaptors, enzymes and chaperones (Figure 2).

The process of TGFβ receptor internalization is complicated and provides characteristic inputs to the EMT response. In other words, the adaptor protein ShcA (specifically its p52 isoform) associates with TGFβRI and shifts the ligand-receptor complex on the cell surface towards cholesterol-enriched microdomains that are internalized in caveolin-coated pits; in this manner, ShcA partitions the TGFβ receptors away from the clathrin-coated pits, thus prohibiting the activation of Smad signaling and reducing the potency of TGFβ to induce EMT [88]. It will be interesting to analyze biological conditions that regulate the recruitment and/or activity of ShcA to the TGFβ receptor during the onset of EMT or MET. In agreement with the previous mechanism, the PDZ domain scaffolding protein, syntenin, can suppress caveolae-dependent TGFβ receptor internalization, promoting Smad-mediated signaling and downstream EMT responses [89]. Mechanistically, syntenin exhibits specificity towards the TGFβRI and interferes with the direct association between TGFβRI and caveolin-1, an integral caveolar membrane protein enriched in these cholesterol-rich domains of the plasma membrane. On the other hand, the presence of the lipid raft domain protein, flotillin-2, on the surface of nasopharyngeal cancer cells correlates with enhanced invasiveness and metastatic potential, which is attributed to enhanced TGFβ signaling causing Src tyrosine kinase activation and β-catenin mobilization to the nucleus, supporting an EMT response in this tumor cell type [90]. The cell type specificity of these various signaling mechanisms that affect the mode of internalization of the TGFβ receptors may reflect the lipid and cholesterol composition of the individual epithelial cell type, which is directly linked to the metabolic activity of the cell.

TGFβ receptor internalization via clathrin-coated pits leads to Smad2 and Smad3 protein phosphorylation and activation of their transcriptional activity. Smad3, on one hand, has been firmly linked to the process of EMT, and knocking out Smad3 in various epithelial cell types, including keratinocytes in the mouse skin, it protects these animals from chemically-induced aggressive carcinoma development [91]. Interestingly, the same animals develop spontaneous squamous cell carcinoma, which illustrates the dual action of the TGFβ signaling pathway during cancer progression. A molecular mechanism that can control the EMT response specifically at the level of Smad3 concerns the phosphorylation of its linker domain by various protein kinases, including MAP-kinase members such as JNKs. JNK1 directly associates with Smad3 and phosphorylates its linker, thus enhancing complex formation with Smad4 and transcriptional activity towards many target genes that constitute the EMT response [92]. Overexpression of a mutant Smad3 with phospho-mimetic residues in its linker domain promotes EMT in lung cells [92]. In contrast, an independent study using a Smad3 mutant lacking the four linker phosphorylation sites showed that it is capable of promoting EMT after overexpression in renal epithelial cells, suggesting a negative role for the Smad3 linker in the EMT process [93]. In agreement with the above, tracheal epithelial cells carrying a complete knockout of JNK1, but not of JNK2, show defects in their EMT response to TGFβ, due to the input JNK1 provides to Smad3 and its associated transcriptional partner, the activation protein 1 (AP-1) heterodimeric complex, in the regulation of pro-invasive genes [94]. These examples illustrate the complexity in identifying the specific contribution of Smad3 linker phosphorylation during the EMT response. Accordingly, a recent report illuminated this complexity by developing a large panel of antibodies that recognize specific phosphorylated residues in the linker region of Smad3 and Smad2 and examining them in the process of liver fibrosis that is characterized by EMT and eventually HCC [95]. According to these studies, Smad2 linker phosphorylation (on residues 245, 250 and 255) correlates with fibrotic EMT, while Smad2 (on residue 220) and Smad3 (on residue 179) linker phosphorylation correlate with the invasive behavior of fibrotic liver cells that progress to carcinoma development. These findings emphasize a potential difference between Smad2 and Smad3 during the course of EMT that is also supported by studies in the keratinocyte-specific Smad2 knockout mouse model, which in contrast to the Smad3 knockout, exhibits a stronger carcinogenic response and signs of EMT following treatment with chemical carcinogens [91].

Silencing of Smad2 or the endocytic protein Smad anchor for receptor activation (SARA), which facilitates Smad2 *C*-terminal phosphorylation by TGFβRI in clathrin-coated endosomes [96], enhanced EMT in renal epithelial cells. In the case of SARA silencing, Smad2 proteasomal degradation mediated by the Smad ubiquitylation regulatory factor 2 (Smurf2) was promoted [96]. Smurf2 is therefore a negative regulator of TGFβ-induced EMT. The enzymatic activity of Smurf2 is induced by sumoylation [97]. TGFβ signaling downregulates the sumo ligase protein inhibitor of activated STAT (PIAS) 1, which is compatible with a mechanism whereby TGFβ attempts to silence its negative regulators; in addition to Smurf2, PIAS1 sumoylates the transcriptional repressor SnoN, which inactivates nuclear Smad complexes [98]. Thus, reduction in PIAS1-dependent sumoylation permits the ubiquitylation and degradation of SnoN and inactivates Smurf2; in this manner, Smad complexes are freer to act and regulate genes critical for the EMT response [97,98]. A second ubiquitin ligase, tripartite motif-containing 33 (TRIM33), is activated by as yet unknown mechanisms and then targets Smad4 to negatively regulate the nuclear Smad complexes, thus antagonizing TGFβ-induced EMT [99]. Smad3 poly-ubiquitylation and proteasomal degradation can also be enhanced by the presence of the tumor suppressor protein ductal epithelium–associated ring chromosome 1 (DEAR1)/tripartite motif-containing 62 (TRIM62); tumor cells that carry loss of function mutations in DEAR1/TRIM62 exhibit enhanced Smad3 and corresponding TGFβ signaling levels, promoting the expression of Snail1 and Snail2 and downstream EMT [100].

Smad3 interacts and cooperates with HDAC6 during EMT in A549 lung adenocarcinoma cells; HDAC6 is well known as a regulator of tubulin acetylation causing microtubule instability and blocking cell migration [101]. In this specific mechanism, HDAC6 seems to play a new role by regulating Smad3 activity, but the link between this mechanism and the function of microtubules has not yet been examined. Nevertheless, a strong correlation has been reported whereby epithelial cells exhibit highly acetylated α-tubulin levels, whereas TGFβ signaling activates HDAC6 and the resulting mesenchymal cells express non-acetylated α-tubulin [102]. Looking at the overall contribution of Smad proteins to the EMT process, one can suggest that Smad2 may protect from EMT, whereas Smad3 promotes EMT, however, this simple model seems to depend on the phosphorylation status of the linker domain of these R-Smads, suggesting a higher level of complexity is involved in the function of these two Smad proteins during EMT.

The MAP-kinase kinase, kinase TAK1, receives signals from the oligomeric TGFβ receptors via ubiquitylation catalyzed by TRAF6; TAK1 then phosphorylates and activates several downstream protein kinases such as p38 and JNK, which are required for the EMT response [15,103]. In mesothelial cells, TAK1 provides phosphorylation inputs that activate the AP-1, Smad3 and NF-κB transcriptional regulators during the EMT response of peritoneal fibrosis [104]. TAK1, via p38 MAP-kinase, phosphorylates c-Jun (an AP-1 component), and inactivates glycogen synthase kinase 3β (GSK3β), a negative regulator of Snail1 stability, leading to transcriptional induction and stabilization of Snail1 and thus promoting EMT [105]. On the other hand, and unexpectedly, genetic silencing of TAK1 in squamous cell carcinoma cells promoted EMT and the invasive properties of these cells as a result of enhanced crosstalk between integrin receptors and the Rac1 small GTPase that induce ROS [106]. This behavior may depend on alternative tumorigenic perturbations that these cells have, such that lowering the expression of TAK1 promotes EMT and tumor aggressiveness. TRAF6 activated by the TGFβ receptors ubiquitylates TGFβRI and activates the plasma membrane-associated proteases TNFα-converting enzyme (TACE) and presenilin-1, which catalyze two subsequent proteolytic cleavages on TGFβRI, one extracellular by TACE and one within the transmembrane domain by presenilin-1. This leads to the release of the extracellular and cytoplasmic domains of TGFβRI; the latter moves to the nucleus and, via cooperation with AP-1, activates transcription of genes such as Snail1 that promote EMT [14,107].

An independent signaling pathway that is activated by TGFβ receptors involves the protein kinase known as mammalian target of rapamycin (mTOR) complex 2 (mTORC2), which directs cytoskeletal reorganization during EMT [108]. Related to the mTOR pathway is the tuberous sclerosis protein TSC1, best known for its regulatory inputs to mTOR signaling activity; TSC1 has now been found to form protein complexes with TGFβRI and Smad2-Smad3, thus promoting receptor–Smad complexes and phosphorylation of the R-Smads [109]. TSC1 was accordingly shown to link protein kinase signaling by Akt to Smad2/3 activation and the promotion of EMT, a function that is distinct from the role of TSC1, together with its related TSC2, in mTOR kinase regulation. The mTORC2 kinase is composed of mTOR, the kinase subunit, and Rictor, the regulatory subunit, which can regulate other protein kinases by forming distinct complexes with them, such as the Rictor–integrin-linked kinase (ILK) complex [110]. Thus, TGFβ activates Akt and mTORC2 activity as described above and also promotes a Rictor–ILK complex. Blocking the catalytic activity of ILK or genetically silencing ILK is sufficient to abrogate most of the EMT responses of mammary cells to TGFβ [110]. By phosphorylating the polarity protein Par6, TGFβRII induces the recruitment of Par6-bound atypical protein kinase C (aPKC) isoforms to the TGFβ receptor, resulting in the reorganization of the actin cytoskeleton and the disassembly of the tight and adherens junction during the EMT response [111]. This mechanism provides coordinate control of epithelial junctional assembly destruction to the formation of the lamelipodia in migratory mesenchymal cells.

Certain protein kinases are strongly upregulated during EMT. One such kinase, Pyk2, whose expression is upregulated during TGFβ-induced EMT, links signaling receptors to the acto-myosin apparatus and promotes cell migration and metastatic outgrowth in breast cancer cell models [112]. In a similar manner, the cyclin-dependent kinase 5 (CDK5) is upregulated in mammary epithelial cells in response to TGFβ and activates focal adhesion kinase (FAK) to promote cell migration [113]. Genetic silencing of CDK5 may thus protect mammary epithelial cells from undergoing EMT.

In addition to kinases, protein phosphatases are also implicated in the EMT response. The Src homology 2 domain-containing protein tyrosine phosphatase 2 (SHP2) promotes EMT when activated, whereas genetic silencing of SHP2, or its pharmacological inhibition, blocks the TGFβ-induced EMT in A549 adenocarcinoma cells [114]. Epithelial cells inactivate SHP2 via the adaptor protein Hook1, which directly binds to SHP2; Hook1 expression is downregulated in mesenchymal cells generated by TGFβ signaling [114]. SHP2 is also relevant to the crosstalk between TGFβ and EGF signaling during EMT, as EGF activates SHP2 activity by recruitment of the adaptor protein GAB1 to the EGF receptor–Grb2 complex [115]. The phosphatase and tensin homologue deleted from chromosome 10 (PTEN) binds to E-cadherin via β-catenin and phosphorylation of the PTEN *C*-terminal tail negatively regulates its phosphatase activity [116]. Thus, in lung cancer cells, TGFβ signaling induces PTEN *C*-terminal domain phosphorylation, and silencing of the phosphatase activity is required for EMT and for lung cancer cell invasion.

Additional cytoplasmic mediators of the EMT response involve the redox protein, thioredoxin, whose expression is upregulated during EMT and in metastatic cancers, whereas inhibition of its upstream enzyme, thioredoxin reductase 1, blocked TGFβ-mediated EMT [117]. The chaperone, clusterin, is transcriptionally induced by TGFβ via the activity of Twist1 and is required for the EMT and metastatic dissemination of prostate cancer cells [118]. Recent unbiased screens at a proteome-wide level promise an even more complete understanding of the molecular players involved downstream of TGFβ that play important roles in the EMT response. A phosphoproteomic screen based on the SILAC technology and mass spectrometric analysis has revealed several phosphoproteins whose levels change critically during the response of keratinocytes to TGFβ. Many of these phosphoproteins have known functional properties that are linked to the differentiation of epithelial cells or the fibrotic response, including ECM proteins [119]. A more focused screen has analyzed only the tyrosine-phosphorylated proteins in lung adenocarcinoma cells responding to TGFβ and undergoing EMT [120]. Both growth factor receptors (e.g., c-Met) and regulatory cytoplasmic proteins (e.g., tensin) were identified in this screen on the basis of having their phospho-tyrosine content increased at specific tyrosine residues. These screens have provided interesting biomarkers that recapitulate previous findings from the in vitro culture of specimens from lung cancer patients [120].

Mathematical analysis of mRNA profiles from lung or pancreatic epithelial cells responding to TGFβ that were divided into stages of initiation, maturation and stabilization, has provided a more quantitative understanding of the EMT response [121]. Based on this work, maturation towards EMT can be explained as a metabolic switch whereby cytosolic ATP levels increase significantly and are probably providing the metabolic energy that is required for the switch, including the engagement of the acto-myosin apparatus during its reorganization. Accordingly, metabolomic analysis of TGFβ-induced EMT has highlighted changes that occur in the enzymes that catalyze lipid biosynthesis and ATP synthesis within mitochondria [122]. In agreement with the mathematical model of EMT [121], TGFβ was shown to suppress enzymes that synthesize lipids from glucose and induce the expression of mitochondrial enzymes of the respiratory chain [122]. Thus, several molecular pathways are involved in the response of epithelial cells to TGFβ as they progress towards the mesenchymal phenotype.

### 3.3. RNA Regulators and Translational Control

Over the last several years, tremendous progress has been made in understanding the role of non-coding RNAs during the process of EMT and their regulation by TGFβ signaling (Figure 2). These include miRNAs and long non-coding RNAs. In addition, major progress has been made in addressing mechanisms of mRNA translation that drive selective synthesis of proteins in an isoform-specific manner. At the post-transcriptional level, regulation of alternative mRNA splicing has become an emerging area of attention in the EMT field. This is best exemplified by the pattern of expression and regulation of the splicing regulators, ESRP1 and ESRP2, which are expressed in epithelial cells but which are downregulated in response to TGFβ and during EMT under the transcriptional repressor activity of ZEB1 and ZEB2, as discussed earlier [63].

Another characteristic case is the regulation of EMT transcription factors by miRNAs downstream of TGFβ signaling. The best example so far is the miR-200 family of miRNAs, whose members show complementarity with and regulate the mRNAs for ZEB1 and ZEB2 [123,124,125]. Since the miR-200s downregulate pro-EMT factors, they act as pro-epithelial mediators, which has been proven by experiments where the miRNAs are overexpressed. Consistently, TGFβ signaling, which promotes the mesenchymal phenotype, downregulates the expression of many of the miR-200 family of miRNAs, including miR-200a, -200b, -200c, -141 and -429 [123,124,125]. In addition to ZEB1/2, miR-200s downregulate expression of the TGFβ2 ligand mRNA during the early phase of the EMT response, thus generating a feedback regulatory loop, whereby TGFβ1 and TGFβ2 suppress miR-200s via binding of ZEB1/2 to the miR-200 silencer elements [123,126]. During the late phase of the EMT response, the miR-200 locus becomes hypermethylated. According to this model, cycles of EMT and MET can be regulated by the input of TGFβ and ZEB1/2 (EMT) followed by the input of miR-200s (MET), which suggests that temporal control mechanisms must exist that define the extent of the EMT or MET period in the life of epithelial cells [127]. Mathematical modeling and high throughput screens of gene expression during EMT and MET have repeatedly identified the importance of the miR-200-ZEB1/2 loop, but also another regulatory loop involving Snail1 and miR-34, which negatively regulate each other [128]. Furthermore, miR-200b has been shown to target and downregulate Smad2 mRNA in intestinal epithelial cells [129]. Similar to the miR-200/ZEB regulatory loop, miR-203 has been shown to target Snail2, and TGFβ signaling induces expression of Snail2, whereas it represses expression of miR-203 [130]. Regulation of the miR-200 family by TGFβ involves the Akt protein kinase isoforms, Akt1 and Akt2 [131]. According to this mechanism, Akt1 blocks the downregulation of miR-200 by TGFβ, whereas Akt2 is required for this downregulation; it remains to be explained how the two specific Akt isoforms become implicated in this regulatory mechanism. In simple terms, one can conclude that TGFβ, via Akt2, provides repressive signaling inputs to miR-200 family members so that their expression is downregulated.

MiR-200 family members regulate additional mRNAs, including the silent information regulator 1 (SIRT1), an established nicotinamide-dependent histone deacetylase. The pro-tumorigenic role of SIRT1 has been attributed to the downregulation of miR-200a by TGFβ signaling, thus permitting SIRT1 overexpression and release of its oncogenic activity [132]. However, it should be kept in mind that opposing evidence in breast cancer cells suggests that SIRT1 protects from EMT, and metastasis and SIRT1 downregulation is required for the progression to EMT and invasiveness [133]. The anti-EMT action of SIRT1 has been suggested to involve de-acetylation of Smad4, which then limits the transcriptional activity of TGFβ signaling. On the other hand, miR-200 expression is regulated by additional mechanisms that do not involve TGFβ directly; epigenetic silencing of the miR-200 locus is frequently observed in carcinomas [132] and PDGF-D provides independent signaling input, causing miR-200 downregulation during EMT [134].

Several other miRNAs that regulate the expression levels of TGFβ signaling components have been linked to the regulation of EMT, MET and stemness in normal embryonic or tumor cells. MiR-99a and -99b are induced by TGFβ signaling and then negatively regulate the basal TGFβ pathway by affecting R-Smad *C*-terminal phosphorylation by the type I receptor [135]. It is possible that miR-99a/b also target the mTOR kinase and thus mediate its effects on EMT, but this remains to be elucidated. In colorectal cancer, miR-187 expression is frequently downregulated; miR-187 can suppress basal TGFβ/Smad signaling and thus is protective from the EMT response [136]. The direct targets of miR-187 include the homeobox transcription factor, Sox4, which also contributes to EMT, and so, the effects of miR-187 against TGFβ signaling and Sox4 may explain why miR-187 is downregulated in colorectal cancer cells. The miR-302 and miR-372 downregulate the expression of TGFβRII and Smad2 and suppress TGFβ signaling in normal somatic fibroblasts, thus enforcing an MET response, which is important for the reprogramming and induction of pluripotent stem cells [137]. The mechanism of reprogramming during induced pluripotency by miR-302 also involves downregulation of intracellular BMP inhibitors and activation of BMP signaling, which contributes to the pluripotency of the induced stem cells [138]. However, miR-302 and miR-372 also downregulate other important mediators of the EMT and of tumor cell invasiveness, including the small GTPase RhoC, the transcription factor ZEB1 and fibronectin [137,139]. In addition, a more direct regulatory input towards transcription factors that maintain the epithelial phenotype has been revealed based on the study of miR-155, which downregulates the pro-epithelial CCAAT-enhancer-binding protein β (C/EBPβ), a direct and positive regulator of epithelial genes such as E-cadherin or tight junction integral proteins [140]. Thus, by acting on C/EBPβ, miR-155 facilitates the EMT process induced by TGFβ.

In addition to miRNAs, an emerging group of EMT regulators are the long non-coding RNAs (lncRNAs), whose function can either be positive or negative with respect to the mechanism at stake. TGFβ signaling regulates the expression of several hundred such lncRNAs during EMT [141]. Three recent examples illustrate these mechanisms well. Malat1 is an lncRNA that is transcriptionally induced by TGFβ signaling during EMT of bladder cancer cells [142]. Malat1 then forms ribonucleoprotein complexes with the transcriptional co-repressor protein suppressor of zeste 12 (Suz12), which plays a role in the E-cadherin to N-cadherin transcriptional switch. In HCC cells, the lncRNA-induced by TGFβ (lncRNA-ATB) acts as a molecular “sponge” that hybridizes to miRNAs of the miR-200 family and inhibits them from targeting ZEB1/2 [143]. In this manner, lncRNA-ATB promotes EMT downstream of TGFβ as it protects the stability and abundance of ZEB1/2, but it is also implicated in HCC metastasis since it upregulates IL-11 mRNA [143]. Lastly, the lncRNA-HIT (HOXa transcript induced by TGFβ) is also required for TGFβ-mediated EMT and tumor cell invasion [141]. LncRNA-HIT may act in a similar manner to malat1 because lncRNA-HIT is involved in the downregulation of E-cadherin during EMT, however, the exact mechanism of action of lncRNA-HIT remains to be elucidated. It is worth noting that in addition to lncRNAs that act as miRNA “sponges”, protein-coding mRNAs may also provide the same type of function, which may be exerted independent from their potential to be translated into proteins. Such mRNAs have been termed competing endogenous RNAs (ceRNAs) and several of these ceRNAs are implicated in cancer progression, including the process of EMT [144]. However, no clear example of a ceRNA that is involved in TGFβ-induced EMT has so far been presented.

Intimately linked to the mode of action of miRNAs that regulate the stability or translation of mRNAs, additional mechanisms that control the expression of mRNAs at the ribosomal translation level are relevant. Two important regulators of TGFβ-induced EMT, the regulator of endocytic trafficking, Dab2, and the cytokine, ILEI, contain conserved regulatory RNA sequences in their 3′untranslated regions (3′UTRs), which are recognized by the translational regulatory complex of the elongation factor, 1A1 (eEF1A1), together with the heterogeneous ribonucleoprotein, E1 (hnRNPE1) [145,146]. When the Dab2 or ILEI mRNAs are bound to hnRNPE1/eEF1A1 complexes, ribosomal translation is stalled along their mRNAs and protein synthesis is suppressed. TGFβ inactivates this negative translational control by triggering the Akt2 protein kinase to phosphorylate hnRNPE1, causing the dissociation of the complex from the target mRNAs and induction of their translation [145]. The same translational complex regulates inhibin βA protein synthesis, which makes part of the activin ligand that also contributes to EMT and tumor cell invasiveness [147]. Another translationally regulated mRNA that contributes to the EMT induced by TGFβ encodes for neuropilin-2, a well-studied receptor for semaphorins and a co-receptor for various other signaling pathways [148]. Induction of neuropilin-2 levels in lung cancer cells contributes to EMT, invasiveness and metastasis [148]; furthermore, neuropilin-2 is highly expressed in HCCs with mesenchymal features and also contributes to the invasiveness of these tumor cells [149]. These mechanisms apply to an even larger cohort of mRNAs that are translationally regulated during TGFβ signaling and the onset of EMT, as identified by unbiased screens of RNA immunoprecipitation followed by sequencing of the RNA [150] or by microarray analysis of translationally regulated (polysome-bound) mRNAs [39]. A larger focus on the post-transcriptional mechanisms that control EMT promises to provide a rich output of molecular pathways that control the multiple steps in mesenchymal transition.

### 3.4. Nuclear Regulators: Transcription and Chromatin Factors

The relevant transcriptional mechanisms that contribute to EMT and act under the control of TGFβ signaling have been presented earlier in this review (Figure 2). Continuous research efforts aim to elucidate at a deeper level the details of how chromatin and transcription factors cooperate in order to establish EMT or MET, and the functional outcome of such nuclear activities is often collectively called reprogramming [8]. A central feature of the basic EMT-TFs is their ability to repress epithelial genes such as E-cadherin and to induce the expression of mesenchymal genes or of other genes implicated in cell invasiveness [42]. In addition, many of the EMT-TFs, once their levels are induced or stabilized, seem to upregulate the expression of other EMT-TFs, thus forming positive feed-forward regulatory loops [151,152,153,154]. While most studies have focused on the dependence of EMT on the regulatory loops between Snail1, Snail2, ZEB1, ZEB2 and Twist1, unbiased mathematical modeling of the EMT response in lung adenocarcinoma A549 cells has also revealed a similar interdependent network that includes the transcription factors HNF4α, Ets2 and JunB [155]. These three transcription factors positively auto-induce their own expression and each of them induces the expression of the other, establishing a positive regulatory triangular network that eventually establishes the EMT, including its hallmark E-cadherin to N-cadherin switch [155]. Thus, EMT is not driven by a single “master” transcription factor, but rather the EMT program is executed by the shared or convergent activities of many such transcription factors. Interestingly, TGFβ signaling provides inputs to many of these transcription factors by controlling their transcription, the stability or translation of their mRNA via miRNAs, their protein stability, subcellular localization and formation of complexes with other transcriptional regulators, central among which stand the Smad proteins [7]. For these reasons, genetic silencing using RNAi [156] or genetic ablation using the CRSPR/Cas9 system [157] of a single EMT-TF, such as Snail1, is not sufficient to revert mesenchymal cells that are generated by the action of TGFβ back to the epithelial phenotype, as several other EMT-TFs remain active and compensate for the loss of one of them.

A rather comprehensive signaling pathway that links TGFβ/Smad activity to the basic EMT-TFs is currently understood. According to this pathway, TGFβ transcriptionally induces the chromatin architectural transcription factor high mobility group A2 (HMGA2), as well as Snail1 and ZEB1 [7]. Smads bind directly to HMGA2 and activate the enhancer elements of Snail1, whereas HMGA2 bound to yet uncharacterized co-factors activates the enhancer on the Twist1 gene [154,156]. HMGA2 also binds and transactivates the Snail2 gene during TGFβ-induced EMT [158]. Smads and Snail1 form protein complexes and together are recruited to the silencer elements of the epithelial genes, E-cadherin, occludin and coxsackie and adenovirus receptor (CAR) [159]. The latter provides a primary signal for the onset of transcriptional repression of these genes. Long-term TGFβ signaling causes HMGA2 to recruit the DNA methyltransferase, DNMT3A to the E-cadherin gene, establishing a more permanent silencing of the gene via methylation [160]. Transcriptional induction of ZEB1 by TGFβ also depends on the cooperative actions of Snail1 and Twist1, both binding to the ZEB1 regulatory sequences [151]. It is also worth remembering that ZEB1 and ZEB2 are genuine Smad-interacting proteins, and thus elicit their downstream pro-EMT activities in cooperation with Smad proteins [161]. The general mechanism involving HMGA2 has been demonstrated in several different models of EMT ranging from normal mammary epithelial cells to breast cancer, colorectal cancer, bladder and nasopharyngeal cancer [158,162,163,164]. It has also been proven that knocking out HMGA2 in the mouse suppresses tumorigenic spread and metastasis, whereas overexpression of HMGA2 promotes metastasis [165]. In addition, HMGA2 was shown to induce the expression of TGFβRII, thus promoting autocrine TGFβ signaling, which also indicates the importance of feed-forward positive loops in the course of EMT, as TGFβ induces HMGA2, which promotes TGFβ signaling [165].

TGFβ1 induces its cousin, TGFβ3, via a β-catenin/T cell factor 4 (TCF4) complex, whose expression levels and stability are induced by the EMT-TFs, Snail1 and Snail2 [152]. Similarly, in breast cancer, Snail1 and Snail2 induce transcription of TGFβRII and thus enhance responsiveness of mesenchymal cells to TGFβ [166]. The paradigm of cooperation between Smad complexes and β-catenin can be extended to the regulation of specific mesenchymal genes, such as the αSMA gene in lung epithelial cells undergoing fibrotic EMT in response to TGFβ [167], or kidney EMT which depends on the formation of β-catenin/Smad3 complexes that are independent from the conventional β-catenin/LEF1 transcriptional complex [168]. These and more examples underscore the importance of autocrine signaling in the progression of long-term EMT, and also factor in the kinetics of the reverse process (MET), when TGFβ availability drops in the microenvironment [169]. The importance of the temporal action of TGFβ and the establishment of autocrine loops is best described during the course of reprogramming somatic cells to embryonic stem cells via consecutive periods of EMT and MET, the former driven by TGFβ/nodal signaling and the latter by BMP signaling [170].

HMGA2 and Smads are not alone in promoting EMT-TF expression during EMT. In breast EMT, c-Myc and Smads form complexes to transactivate Snail1 expression [171]. In prostate EMT, TGFβ induces Snail2 expression by downregulation of the transcription factor, Krüppel-like factor 4 (KLF4), via proteasomal degradation [172]. In this example, KLF4 and the transcription factor, FOXOA1, protect the epithelial phenotype, while TGFβ signaling suppresses the activities of these transcription factors by promoting the action of Snail2 in the prostate. The KLF family member, KLF17, also promotes epithelial differentiation and associates with the tumor suppressor actions of TGFβ signaling [173]. Thus, TGFβ, via Smad3, induces KLF17 expression, and KLF17 suppresses tumor growth, providing input to the cytostatic action of TGFβ [173]. However, KLF17 also interacts with Smad3 and in cooperation mediates the transcription of specific metastasis genes, a switch in function that may be relevant to HCC progression but which requires a deeper understanding.

In lung cancer cells, thyroid transcription factor 1 (TTF1) promotes the epithelial phenotype, and its downregulation in response to TGFβ is required for Snail1 and Snail2 induction, so that lung EMT can proceed [174]. In fibrotic lung EMT, the forkhead box M1 (Foxm1) protein contributes to the EMT by transactivating the Snail1 gene under conditions of exposure to radiation, which induces lung fibrosis [175]. In HCC, TGFβ mediates Snail1 induction with the assistance of the glioma-associated oncogene 1 (Gli1), which is known to act downstream of the sonic hedgehog pathway [176]. In Ras-transformed breast epithelial cells a similar mechanism operates; TGFβ promotes a switch in the binding of PIAS3 from STAT3 to Smad3, releasing the transcriptional activity of STAT3, which induces the Snail1 gene to promote EMT [177]. Alternatively, TGFβ induces the transcription factor ATF3, in order to transactivate Snail1, Snail2 and Twist1, promoting EMT in Ras-transformed breast cancer cells [178]. Induction of Twist1 in prostate EMT similarly depends on a different transcriptional constellation downstream of TGFβ, namely, on phosphorylated STAT3 and on newly synthesized and proteolytically stabilized hypoxia inducible factor 1α (HIF1α), which bind as a complex to the Twist1 gene to transactivate its transcription [179]. In hamster carcinoma cells, TGFβ induces the small protein p12CDK2-AP1, which in turn induces Twist2 expression [180]. In pancreatic cancer, the inhibitor of NF-κB kinase α (IKKα) forms complexes with Smads and transactivates the Snail1 and Snail2 genes that elicit EMT [181], whereas the Ras oncoprotein promotes Erk MAP-kinase signaling that also contributes to Snail1 induction [182]. Thus, it is evident that Smad complexes, upon activation by TGFβ, make associations with a large variety of transcription factors in order to induce expression of the EMT-TFs. The Smad co-transcription factors may either depend on the cell type where the EMT takes place or, alternatively, on the signaling input or oncogenic stimulus that operates in the cell.

The EMT-TFs not only promote EMT and cell invasiveness but also provide signals that affect the cell cycle and/or cell survival. Accordingly, TGFβ-induced Snail1 suppresses the liver cell death response to TGFβ and helps cells survive and eventually undergo the EMT [183]. However, studies in the context of pancreatic tumor progression provide a more complex picture about the role of Snail1 and EMT with respect to programmed cell death [184]. In pancreatic adenocarcinoma where the TGFβ pathway is intact and the cells express Smad4, TGFβ induces Snail1 and EMT; however, under the coordinate transcriptional regulation between Smads and Snail1, the epithelial transcription factor, KLF5, becomes repressed. In addition, TGFβ/Smad signaling induces expression of the homeobox transcription factor, Sox4, which enforces apoptosis despite the presence of Snail1 [184]. The latter event, a co-occurrence of EMT and apoptosis, has been termed “lethal EMT”. Interestingly, when pancreatic tumors mutate their Smad4 gene and thus lose its expression, TGFβ signaling fails to induce Snail1 or EMT, however, it can induce Sox4 expression; the coordinate action of Sox4 and the pre-existing KLF5 promotes tumorigenesis [184]. It is interesting to consider whether this mechanism applies to other epithelial cancers where Smad4 function is lost (e.g., colorectal cancer). EMT studies in breast cancer have indicated that, similar to the pancreatic cancer example, TGFβ-induced Sox4 expression positively contributed to the EMT and cell survival [185,186]. This effect was, at least in part, exerted via transcriptional upregulation of the Polycomb group histone methyltransferase, Ezh2, whose action is important for the chromatin remodeling that takes place during EMT [185]. Independent studies in breast cancer EMT have indicated that TGFβ-induced Sox4 contributes mainly to the transcriptional upregulation of mesenchymal genes, such as N-cadherin and vimentin, via direct binding of Sox4 to the promoters of these genes, however, the exact transcriptional mechanism has not been clarified yet [187].

An additional set of transcription factors can be induced by TGFβ signaling and contributes to the EMT, however, the link between these proteins and the established EMT-TFs remains to be examined. These include the paired-related homeobox 2 (PRRX2) factor whose upregulation during TGFβ-induced EMT transactivates pro-invasive genes such as the tissue plasminogen activator (tPA) [188]. In breast cancer cells, TGFβ also induces expression of the proto-oncogene c-Myb, which then contributes to the EMT and the invasive gene program [189].

Alternative transcriptional pathways that protect cells from undergoing EMT or induce MET also play important roles during metastatic dissemination. The case of the KLF family proteins was presented earlier. In addition, while TGFβ downregulates the inhibitor of differentiation 2 (ID2) in order to elicit EMT [190,191], BMP signaling promotes ID2 expression and mediates MET [190]. Important transcription factors that mediate the TGFβ/Smad signal that represses the ID2 gene are JunB and Mad [192,193]. In agreement with the above model, downregulating BMP signaling is required for the effective proceeding of EMT. Accordingly, TGFβ signaling, via crosstalk with oncogenic Ras and transcriptional input from the AP-1 complex, induces expression of the transcriptional repressor B lymphocyte-induced maturation protein (Blimp-1), also known as positive regulatory domain zinc finger protein 1 (PRMD1) in breast cancer cells [194]. Blimp-1/PRMD1 then represses the BMP5 gene and thus facilitates the EMT. In addition, Blimp-1/PRMD1 induces Snail1 expression and thus provides a major transcriptional input to the EMT [194]. A transcriptional scenario involving the ID1 protein presents an interesting example on the interdependence of EMT and MET [195]. When TGFβ induces EMT in breast cancer cells, it also induces ID1 expression, which has been linked to stem-like features of these tumor cells [195]. Upon metastatic dissemination in recipient mice, the metastatic breast cancer cells require ID1 to inactivate the transcriptional activity of Twist1 before MET can occur [195]. This mechanism illustrates the alternate switches that tumor cells follow during cancer progression and it also leaves open the question of whether sustained BMP signals are also required for the action of ID1 against Twist1 during metastatic MET.

Another protein related to the IDs is the maternal ID-like molecule (Maid), which is transcriptionally induced by TGFβ at a late phase of the response, and plays a selective role in limiting the pro-migratory response of epithelial cells to TGFβ [196]. The transcription factor grainyhead-like-2 (GRHL2) is involved in the wound-healing response and has been demonstrated to protect from EMT in breast cancer cell models [197,198]. GRHL2 expression is usually low in breast cancers that exhibit features of EMT, such as the claudin-low subclass, but when it is overexpressed it can suppress TGFβ-induced EMT [197]. During the MET program induced by GRHL2 overexpression, Smad and ZEB1 genes were repressed, whereas BMP2 and miR-200b/c genes were transcriptionally induced. GRHL2 represses ZEB1 expression by interfering with some of the positive regulators of ZEB1 expression, including Six1; on the other hand, during EMT induced by TGFβ and Wnt signaling, ZEB1 represses GRHL2 expression and thus removes another mediator of the epithelial phenotype [198]. During normal mammary gland morphogenesis, EMT is followed by periods of epithelialization (or MET), which is partially mediated by the expression of the transcription factor, ovo-like zinc finger 2 (Ovol2) [199]. When Ovol2 is missing, the TGFβ-induced cytostatic effect is suppressed and the mammary epithelium exhibits an uncontrolled EMT, whereas in the presence of Ovol2, many of the EMT-TF genes remain transcriptionally silent and TGFβ signaling exhibits mainly its cytostatic function in the developing mammary epithelium [199]. Thus, regulation of Ovol2 expression and function can mediate cycles of EMT and MET during normal ductal morphogenesis.

The large number of transcriptional examples enlisted so far need to also be complemented by a look at the epigenetic or chromatin-based modifications that accompany changes in gene expression during EMT. Interestingly, at a genome-wide level, the mesenchymal phenotype correlates with a DNA state of high methylation, whereas the epithelial phenotype correlates with a lower level of DNA methylation; TGFβ signaling via Smads induces expression of the DNMT1 methyltransferase, which contributes to the global de novo methylation during EMT [200]. One example of a gene target that is sensitive to such de novo methylation is the Twist1 gene [201]. Cooperating with DNA methylation, histone modifications also accompany the EMT process, such as low dimethylation of histone H3 lysine 9 (a repressive chromatin mark) and high trimethylation of H3 lysine 4 (a positive chromatin mark), which can be mediated by the activity of the Lsd1 demethylase, another factor that is induced by TGFβ during EMT [202]. It should be noted that Lsd1 presents dual actions and its function mainly links to H3 demethylation and heterochromatin assembly, but Lsd1 can also mediate H3 lysine 4 demethylation, which occurs during euchromatin assembly [202]. In addition, the methyltransferase jumonji and AT-rich interaction domain-containing 2 (JARID2) is induced by TGFβ signaling in lung or colon carcinoma cells. JARID2 contributes to the methylation of H3 lysine 27, a repressive chromatin mark that is associated with epithelial genes such as E-cadherin and the miR-200 family that are shut off during EMT [203]. Another gene that is affected by such epigenetic regulation is the metastasis suppressor 30 kDa HIV-1 Tat-interacting protein (TIP30), whose expression is repressed by TGFβ signaling via enhanced hypermethylation of the TIP30 regulatory sequences in esophageal carcinoma cells [204]. One aspect of such epigenetic control is the regulation of TGFβ signaling components such as the Smad2 and Smad3 genes, whose expression is negatively regulated by HDAC1; during lung cancer cell EMT, profilin-2 directly binds to HDAC1 and sequesters it from its nuclear–chromatin targets, resulting in the upregulation of Smad2 and Smad3, which can then contribute to the EMT response [205]. Smad3 expression during EMT is also dependent on specific transcriptional inputs such as the one provided by the Pre-B-cell leukemia homeobox (Pbx)-regulating protein-1 (PREP1). PREP1, together with the AP-1 member, FRA1, transactivate an enhancer of the Smad3 gene, providing a more sensitive response of lung adenocarcinoma cells to TGFβ [206]. The details of the chromatin-based (or epigenetic) regulation that is mediated by the plethora of transcription factors operating downstream of TGFβ during EMT, and the individual target genes that are accordingly modified, await future analysis.

## 4. Development of Drugs that Block TGFβ-Induced EMT

The rich evidence that has accumulated through the years on the various mechanisms that mediate EMT after TGFβ stimulation, has logically led to many attempts to identify chemical modifiers of this process that could be developed into clinically useful anti-metastatic drugs. This is a rapidly evolving area of research and here we list several of the most recent efforts to generate anti-EMT inhibitors. The ability of genuine TGFβ receptor kinase inhibitors or anti-TGFβ ligand traps to block EMT and affect metastasis or fibrosis has been reviewed exhaustively elsewhere [207]. It is worth mentioning, though, that recent attempts to combine TGFβ receptor inhibitors with classical anti-cancer drugs, such as paclitaxel, have generated new enthusiasm based on the efficiency of these combinations in blocking EMT, metastasis to the lung and stem-like features of breast cancer cell models [208].

Based on previously established knowledge about the peroxisome proliferator-activated receptor γ (PPARγ), which acts against tissue fibrosis, recent work has established an anti-EMT action for PPARγ in lung epithelial cells [209]. The available chemical agonists of PPARγ, such as troglitazone or rosiglitazone, inhibit TGFβ-induced EMT of the alveolar cells. However, whether these established PPARγ agonists block TGFβ-induced EMT by activating PPARγ functions specifically, remains unclear [209]. In an independent lung model system of EMT using the A549 adenocarcinoma cells, an unbiased screen for chemical inhibitors that would not block canonical TGFβ signaling identified methacycline to be effective in preventing many of the molecular features of EMT [210]. Methacycline effectively reduced phenotypic features of bleomycin-induced lung fibrosis and its action appears to target the epithelial cells, but not inflammatory cells such as macrophages. The established anti-fibrotic agent, hydrogen sulfide, was also recently proven to block the TGFβ-induced EMT of kidney epithelial cells by suppressing the expression levels of TGFβ receptors and also by attenuating β-catenin activation by TGFβ [211]. The effects of hydrogen sulfide could be reduced upon inhibition of the Erk/MAP-kinase pathway or by employing dominant negative mutants of β-catenin.

The established modulator of p53 stability and biological activity, nutlin-3, also blocks TGFβ-induced EMT in carcinoma cell lines, even when these cells lack p53 or carry mutant forms of p53. Surprisingly, nutlin-3 was found to affect the phosphorylation levels of Smad2 and Smad3, thus blocking downstream Snail1/2 activation and suggesting that nutlin-3 could act as a genuine TGFβ receptor inhibitor [212]. The inhibitor of NF-κB signaling, disulfiram, which is used clinically in the treatment of alcohol-dependent tissue damage, has also been shown to inhibit TGFβ-induced EMT and primary tumor growth of breast cancer cell models [213]. The mechanism by which disulfiram blocks NF-κB and downstream Snail1 activity requires further characterization in order to understand the mode of action of this compound during EMT. Based on the epigenetic reprogramming that accompanies the EMT, it is logical that general HDAC inhibitors like vorinostat can block TGFβ-mediated EMT in vitro and metastasis of biliary tract cancer cells, while also reducing the chemoresistance developed by such cells [214]. Interestingly, the molecular mode of action of vorinostat appeared to target the nuclear accumulation of Smad4, suggesting that HDACs may regulate the shuttling mechanism of Smad4. The impact of translational control during TGFβ-induced EMT has been presented above; this evidence led to the identification of 4Ei-1, a chemical inhibitor of the translation initiation factor eIF4E, which blocks TGFβ-induced EMT by reducing the pool of Snail1 mRNA which is translated by polysomes at the early onset of the EMT [215]. At the extracellular level, PAI1 has been considered a hallmark molecule that represents activation of TGFβ signaling during tissue fibrosis or cancer. The PAI1 inhibitor SK-216 blocks TGFβ-induced EMT, including PAI1 neosynthesis, fibroblast to myofibroblast terminal differentiation and bleomycin-induced lung fibrosis [216].

In addition, some compounds enriched from herbs or other natural sources have proven effective in blocking TGFβ-induced EMT, which may explain their potency in reducing the metastatic potential of breast cancer cell models in recipient mice [217]. One such compound is curcumin, which has been used in the treatment of various tumors and recently was shown to promote epithelial features in thyroid carcinoma cells by counteracting the actions of TGFβ. The specific mechanism of action for curcumin is unclear but data indicate it may impact on the expression levels or activity of the TGFβ receptors [218]. The vegetable ingredient, sulforaphane, also inhibits TGFβ-mediated EMT in HCC by modulating ROS production, which indicates a novel function for this compound in addition to its established general anti-tumor activity [219]. Independent screens for the action of the proteasome identified protein downregulation of the β2 and β5 subunits of the proteasome during EMT, and highly specific proteasome inhibitors, whose action has been recommended against various cancers, were shown to promote EMT and stem-like features in breast cancer cell models [220]. This new evidence generates some cautionary alarm bells regarding the beneficial use of proteasomal inhibitors in the context of cancer or tissue fibrosis. Thus, current pharmacological approaches actively seek to identify new anti-EMT compounds as well as combinations of compounds that could be used as cocktails to effectively combat the metastatic dissemination of tumor cells derived from a large variety of carcinomas.

## 5. Conclusions

The impact of TGFβ signaling on the regulation of EMT is undoubtedly large. This growth factor not only initiates the process but then establishes a dramatic cellular adaptation that permeates a large number of vital cell biological processes. In this manner, TGFβ promotes EMT in a wide variety of epithelial cell types. The interdependency of TGFβ with subsequent circles of cytokine and growth factor signaling is of primary importance. The delicate networks of small or large non-coding RNAs, together with many signaling enzymes, transcription factors and chromatin remodeling factors, cooperate in the gradual and step-wise establishment of a genome-wide change to the chromatin state that affects a large cohort of genes whose expression is regulated during the EMT. The specific molecular actors that control the epithelial genes and the mesenchymal genes are not yet fully elucidated and future work should identify many of the intricate details of these transcriptional programs. A better understanding of the signaling networks should reveal nodes that may prove sensitive to pharmacological inhibition and assist in the development of drugs that block the EMT, metastasis and tumor-initiating capacity of cancer cells. Future work will allow for the implementation of such knowledge to the development of new anti-cancer therapies based on the extensive molecular paradigm of TGFβ-induced EMT.

## References

[1] Nieto M.A. (2013). Epithelial plasticity: A common theme in embryonic and cancer cells. Science.

[2] Gonzalez D.M., Medici D. (2014). Signaling mechanisms of the epithelial-mesenchymal transition. Sci. Signal..

[3] Van Meeteren L.A., ten Dijke P. (2012). Regulation of endothelial cell plasticity by TGF-β. Cell. Tissue Res..

[4] Kalluri R., Weinberg R.A. (2009). The basics of epithelial-mesenchymal transition. J. Clin. Investig..

[5] Moustakas A., Heldin C.-H. (2007). Signaling networks guiding epithelial-mesenchymal transitions during embryogenesis and cancer progression. Cancer Sci..

[6] Derynck R., Muthusamy B.P., Saeteurn K.Y. (2014). Signaling pathway cooperation in TGF-β-induced epithelial-mesenchymal transition. Curr. Opin. Cell. Biol..

[7] Moustakas A., Heldin C.-H. (2012). Induction of epithelial-mesenchymal transition by transforming growth factor β. Semin. Cancer Biol..

[8] Ye X., Weinberg R.A. (2015). Epithelial-Mesenchymal Plasticity: A Central Regulator of Cancer Progression. Trends Cell Biol..

[9] Thiery J.P., Acloque H., Huang R.Y., Nieto M.A. (2009). Epithelial-mesenchymal transitions in development and disease. Cell.

[10] Moustakas A., Heldin C.-H. (2009). The regulation of TGF-β signal transduction. Development.

[11] ten Dijke P., Arthur H.M. (2007). Extracellular control of TGF-β signalling in vascular development and disease. Nat. Rev. Mol. Cell. Biol..

[12] Travis M.A., Sheppard D. (2014). TGF-β Activation and Function in Immunity. Annu. Rev. Immunol..

[13] Gudey S.K., Wallenius A., Landström M. (2014). Regulated intramembrane proteolysis of the TGF-β type I receptor conveys oncogenic signals. Future Oncol..

[14] Mu Y., Sundar R., Thakur N., Ekman M., Gudey S.K., Yakymovych M., Hermansson A., Dimitriou H., Bengoechea-Alonso M.T., Ericsson J. (2011). TRAF6 ubiquitinates TGF-β type I receptor to promote its cleavage and nuclear translocation in cancer. Nat. Commun..

[15] Sorrentino A., Thakur N., Grimsby S., Marcusson A., von Bulow V., Schuster N., Zhang S., Heldin C.-H., Landström M. (2008). The type I TGF-β receptor engages TRAF6 to activate TAK1 in a receptor kinase-independent manner. Nat. Cell. Biol..

[16] Massagué J. (2008). TGF-β in Cancer. Cell.

[17] Mani S.A., Yang J., Brooks M., Schwaninger G., Zhou A., Miura N., Kutok J.L., Hartwell K., Richardson A.L., Weinberg R.A. (2007). Mesenchyme Forkhead 1 (FOXC2) plays a key role in metastasis and is associated with aggressive basal-like breast cancers. Proc. Natl. Acad. Sci. USA.

[18] Borok Z. (2009). Role for α3 integrin in EMT and pulmonary fibrosis. J. Clin. Investig..

[19] Javelaud D., Mauviel A. (2005). Crosstalk mechanisms between the mitogen-activated protein kinase pathways and Smad signaling downstream of TGF-β: Implications for carcinogenesis. Oncogene.

[20] Sundqvist A., Zieba A., Vasilaki E., Herrera Hidalgo C., Soderberg O., Koinuma D., Miyazono K., Heldin C.-H., Landegren U., ten Dijke P. (2013). Specific interactions between Smad proteins and AP-1 components determine TGF-β-induced breast cancer cell invasion. Oncogene.

[21] Safina A., Ren M.Q., Vandette E., Bakin A.V. (2008). TAK1 is required for TGF-β 1-mediated regulation of matrix metalloproteinase-9 and metastasis. Oncogene.

[22] Eckert M.A., Lwin T.M., Chang A.T., Kim J., Danis E., Ohno-Machado L., Yang J. (2011). Twist1-induced invadopodia formation promotes tumor metastasis. Cancer Cell..

[23] Park J., Schwarzbauer J.E. (2014). Mammary epithelial cell interactions with fibronectin stimulate epithelial-mesenchymal transition. Oncogene.

[24] Nguyen H.L., Kadam P., Cao K., Wu S., Samara G.J., Zhang Q., Zucker S., Cao J. (2016). MT1-MMP Activation of TGF-β Signaling Enables Intercellular Activation of an Epithelial-mesenchymal Transition Program in Cancer. Curr. Cancer Drug Targets.

[25] Chanmee T., Ontong P., Mochizuki N., Kongtawelert P., Konno K., Itano N. (2014). Excessive hyaluronan production promotes acquisition of cancer stem cell signatures through the coordinated regulation of Twist and the transforming growth factor β (TGF-β)-Snail signaling axis. J. Biol. Chem..

[26] Porsch H., Bernert B., Mehic M., Theocharis A.D., Heldin C.-H., Heldin P. (2013). Efficient TGFβ-induced epithelial-mesenchymal transition depends on hyaluronan synthase HAS2. Oncogene.

[27] Leight J.L., Wozniak M.A., Chen S., Lynch M.L., Chen C.S. (2012). Matrix rigidity regulates a switch between TGF-β1-induced apoptosis and epithelial-mesenchymal transition. Mol. Biol. Cell..

[28] Varelas X., Wrana J.L. (2012). Coordinating developmental signaling: Novel roles for the Hippo pathway. Trends Cell Biol..

[29] Tan Z., Lu W., Li X., Yang G., Guo J., Yu H., Li Z., Guan F. (2014). Altered *N*-Glycan expression profile in epithelial-to-mesenchymal transition of NMuMG cells revealed by an integrated strategy using mass spectrometry and glycogene and lectin microarray analysis. J. Proteome Res..

[30] Du J., Hong S., Dong L., Cheng B., Lin L., Zhao B., Chen Y.G., Chen X. (2015). Dynamic Sialylation in Transforming Growth Factor-β (TGF-β)-induced Epithelial to Mesenchymal Transition. J. Biol. Chem..

[31] Hirakawa M., Takimoto R., Tamura F., Yoshida M., Ono M., Murase K., Sato Y., Osuga T., Sato T., Iyama S. (2014). Fucosylated TGF-β receptors transduces a signal for epithelial-mesenchymal transition in colorectal cancer cells. Br. J. Cancer.

[32] Scheel C., Eaton E.N., Li S.H.J., Chaffer C.L., Reinhardt F., Kah K.J., Bell G., Guo W., Rubin J., Richardson A.L. (2011). Paracrine and Autocrine Signals Induce and Maintain Mesenchymal and Stem Cell States in the Breast. Cell.

[33] Steinway S.N., Zanudo J.G., Ding W., Rountree C.B., Feith D.J., Loughran T.P., Albert R. (2014). Network modeling of TGF-β signaling in hepatocellular carcinoma epithelial-to-mesenchymal transition reveals joint sonic hedgehog and Wnt pathway activation. Cancer Res..

[34] Wendt M.K., Smith J.A., Schiemann W.P. (2010). Transforming growth factor-β-induced epithelial-mesenchymal transition facilitates epidermal growth factor-dependent breast cancer progression. Oncogene.

[35] Izumchenko E., Chang X., Michailidi C., Kagohara L., Ravi R., Paz K., Brait M., Hoque M.O., Ling S., Bedi A., Sidransky D. (2014). The TGF-β-miR200-MIG6 pathway orchestrates the EMT-associated kinase switch that induces resistance to EGFR inhibitors. Cancer Res..

[36] Maitah M.Y., Ali S., Ahmad A., Gadgeel S., Sarkar F.H. (2011). Up-regulation of sonic hedgehog contributes to TGF-β1-induced epithelial to mesenchymal transition in NSCLC cells. PLoS ONE.

[37] Johnson J.R., Nishioka M., Chakir J., Risse P.A., Almaghlouth I., Bazarbashi A.N., Plante S., Martin J.G., Eidelman D., Hamid Q. (2013). IL-22 contributes to TGF-β1-mediated epithelial-mesenchymal transition in asthmatic bronchial epithelial cells. Respir. Res..

[38] Pang M.F., Georgoudaki A.M., Lambut L., Johansson J., Tabor V., Hagikura K., Jin Y., Jansson M., Alexander J.S., Nelson C.M. (2016). TGF-β1-induced EMT promotes targeted migration of breast cancer cells through the lymphatic system by the activation of CCR7/CCL21-mediated chemotaxis. Oncogene.

[39] Gotzmann J., Mikula M., Eger A., Schulte-Hermann R., Foisner R., Beug H., Mikulits W. (2004). Molecular aspects of epithelial cell plasticity: Implications for local tumor invasion and metastasis. Mutat. Res..

[40] Lahsnig C., Mikula M., Petz M., Zulehner G., Schneller D., van Zijl F., Huber H., Csiszar A., Beug H., Mikulits W. (2009). ILEI requires oncogenic Ras for the epithelial to mesenchymal transition of hepatocytes and liver carcinoma progression. Oncogene.

[41] van Zijl F., Mair M., Csiszar A., Schneller D., Zulehner G., Huber H., Eferl R., Beug H., Dolznig H., Mikulits W. (2009). Hepatic tumor-stroma crosstalk guides epithelial to mesenchymal transition at the tumor edge. Oncogene.

[42] Nieto M.A. (2011). The ins and outs of the epithelial to mesenchymal transition in health and disease. Annu. Rev. Cell Dev. Biol..

[43] Wendt M.K., Taylor M.A., Schiemann B.J., Schiemann W.P. (2011). Down-regulation of epithelial cadherin is required to initiate metastatic outgrowth of breast cancer. Mol. Biol. Cell..

[44] Viloria-Petit A.M., Wrana J.L. (2010). The TGF-β-Par6 polarity pathway: Linking the Par complex to EMT and breast cancer progression. Cell. Cycle.

[45] Schneider D.J., Wu M., Le T.T., Cho S.H., Brenner M.B., Blackburn M.R., Agarwal S.K. (2012). Cadherin-11 contributes to pulmonary fibrosis: Potential role in TGF-β production and epithelial to mesenchymal transition. FASEB J..

[46] Sancisi V., Gandolfi G., Ragazzi M., Nicoli D., Tamagnini I., Piana S., Ciarrocchi A. (2013). Cadherin 6 is a new RUNX2 target in TGF-β signalling pathway. PLoS ONE.

[47] Guan F., Schaffer L., Handa K., Hakomori S.I. (2010). Functional role of gangliotetraosylceramide in epithelial-to-mesenchymal transition process induced by hypoxia and by TGF-β. FASEB J..

[48] Whiteman E.L., Liu C.J., Fearon E.R., Margolis B. (2008). The transcription factor snail represses Crumbs3 expression and disrupts apico-basal polarity complexes. Oncogene.

[49] Yu L., Liu X., Cui K., Di Y., Xin L., Sun X., Zhang W., Yang X., Wei M., Yao Z., Yang J. (2015). SND1 Acts Downstream of TGF-β1 and Upstream of Smurf1 to Promote Breast Cancer Metastasis. Cancer Res..

[50] Kong W., Yang H., He L., Zhao J.J., Coppola D., Dalton W.S., Cheng J.Q. (2008). MicroRNA-155 is regulated by the transforming growth factor β/Smad pathway and contributes to epithelial cell plasticity by targeting RhoA. Mol. Cell. Biol..

[51] Zhou Q., Fan J., Ding X., Peng W., Yu X., Chen Y., Nie J. (2010). TGF-β-induced MiR-491–5p expression promotes Par-3 degradation in rat proximal tubular epithelial cells. J. Biol. Chem..

[52] Pignatelli J., Tumbarello D.A., Schmidt R.P., Turner C.E. (2012). Hic-5 promotes invadopodia formation and invasion during TGF-β-induced epithelial-mesenchymal transition. J. Cell. Biol..

[53] Parvani J.G., Galliher-Beckley A.J., Schiemann B.J., Schiemann W.P. (2013). Targeted inactivation of β1 integrin induces β3 integrin switching, which drives breast cancer metastasis by TGF-β. Mol. Biol. Cell..

[54] Papadimitriou E., Vasilaki E., Vorvis C., Iliopoulos D., Moustakas A., Kardassis D., Stournaras C. (2012). Differential regulation of the two RhoA-specific GEF isoforms Net1/Net1A by TGF-β and miR-24: Role in epithelial-to-mesenchymal transition. Oncogene.

[55] Osborne L.D., Li G.Z., How T., O’Brien E.T., Blobe G.C., Superfine R., Mythreye K. (2014). TGF-β regulates LARG and GEF-H1 during EMT to affect stiffening response to force and cell invasion. Mol. Biol. Cell..

[56] Ngan E., Northey J.J., Brown C.M., Ursini-Siegel J., Siegel P.M. (2013). A complex containing LPP and a-actinin mediates TGFβ-induced migration and invasion of ErbB2-expressing breast cancer cells. J. Cell. Sci..

[57] Haynes J., Srivastava J., Madson N., Wittmann T., Barber D.L. (2011). Dynamic actin remodeling during epithelial-mesenchymal transition depends on increased moesin expression. Mol. Biol. Cell..

[58] Mori M., Nakagami H., Koibuchi N., Miura K., Takami Y., Koriyama H., Hayashi H., Sabe H., Mochizuki N., Morishita R. (2009). Zyxin mediates actin fiber reorganization in epithelial-mesenchymal transition and contributes to endocardial morphogenesis. Mol. Biol. Cell..

[59] Mise N., Savai R., Yu H., Schwarz J., Kaminski N., Eickelberg O. (2012). Zyxin is a transforming growth factor-β (TGF-β)/Smad3 target gene that regulates lung cancer cell motility via integrin α5β1. J. Biol. Chem..

[60] Beach J.R., Hussey G.S., Miller T.E., Chaudhury A., Patel P., Monslow J., Zheng Q., Keri R.A., Reizes O., Bresnick A.R. (2011). Myosin II isoform switching mediates invasiveness after TGF-β-induced epithelial-mesenchymal transition. Proc. Natl. Acad. Sci. USA.

[61] Cantelli G., Orgaz J.L., Rodriguez-Hernandez I., Karagiannis P., Maiques O., Matias-Guiu X., Nestle F.O., Marti R.M., Karagiannis S.N., Sanz-Moreno V. (2015). TGF-β-Induced Transcription Sustains Amoeboid Melanoma Migration and Dissemination. Curr. Biol..

[62] Hinz B., Phan S.H., Thannickal V.J., Galli A., Bochaton-Piallat M.L., Gabbiani G. (2007). The myofibroblast: One function, multiple origins. Am. J. Pathol..

[63] Horiguchi K., Sakamoto K., Koinuma D., Semba K., Inoue A., Inoue S., Fujii H., Yamaguchi A., Miyazawa K., Miyazono K. (2012). TGF-β drives epithelial-mesenchymal transition through δEF1-mediated downregulation of ESRP. Oncogene.

[64] Shirakihara T., Horiguchi K., Miyazawa K., Ehata S., Shibata T., Morita I., Miyazono K., Saitoh M. (2011). TGF-β regulates isoform switching of FGF receptors and epithelial-mesenchymal transition. EMBO J..

[65] Charbonney E., Speight P., Masszi A., Nakano H., Kapus A. (2011). Beta-catenin and Smad3 regulate the activity and stability of myocardin-related transcription factor during epithelial-myofibroblast transition. Mol. Biol. Cell..

[66] Masszi A., Speight P., Charbonney E., Lodyga M., Nakano H., Szaszi K., Kapus A. (2010). Fate-determining mechanisms in epithelial-myofibroblast transition: Major inhibitory role for Smad3. J. Cell. Biol..

[67] Morita T., Mayanagi T., Sobue K. (2007). Dual roles of myocardin-related transcription factors in epithelial mesenchymal transition via slug induction and actin remodeling. J. Cell. Biol..

[68] Järvinen P.M., Laiho M. (2012). LIM-domain proteins in transforming growth factor β-induced epithelial-to-mesenchymal transition and myofibroblast differentiation. Cell. Signal..

[69] Yu Y., Xiao C.H., Tan L.D., Wang Q.S., Li X.Q., Feng Y.M. (2014). Cancer-associated fibroblasts induce epithelial-mesenchymal transition of breast cancer cells through paracrine TGF-β signalling. Br. J. Cancer.

[70] Andarawewa K.L., Erickson A.C., Chou W.S., Costes S.V., Gascard P., Mott J.D., Bissell M.J., Barcellos-Hoff M.H. (2007). Ionizing radiation predisposes nonmalignant human mammary epithelial cells to undergo transforming growth factor β induced epithelial to mesenchymal transition. Cancer Res..

[71] Kim H., Choi J.A., Kim J.H. (2014). Ras promotes transforming growth factor-β (TGF-β)-induced epithelial-mesenchymal transition via a leukotriene B4 receptor-2-linked cascade in mammary epithelial cells. J. Biol. Chem..

[72] Mele V., Muraro M.G., Calabrese D., Pfaff D., Amatruda N., Amicarella F., Kvinlaug B., Bocelli-Tyndall C., Martin I., Resink T.J. (2014). Mesenchymal stromal cells induce epithelial-to-mesenchymal transition in human colorectal cancer cells through the expression of surface-bound TGF-β. Int. J. Cancer.

[73] Thakur A.K., Nigri J., Lac S., Leca J., Bressy C., Berthezene P., Bartholin L., Chan P., Calvo E., Iovanna J.L. (2016). TAp73 loss favors Smad-independent TGF-β signaling that drives EMT in pancreatic ductal adenocarcinoma. Cell. Death Differ..

[74] Labelle M., Begum S., Hynes R.O. (2011). Direct signaling between platelets and cancer cells induces an epithelial-mesenchymal-like transition and promotes metastasis. Cancer Cell..

[75] Del Pozo Martin Y., Park D., Ramachandran A., Ombrato L., Calvo F., Chakravarty P., Spencer-Dene B., Derzsi S., Hill C.S., Sahai E. (2015). Mesenchymal Cancer Cell-Stroma Crosstalk Promotes Niche Activation, Epithelial Reversion, and Metastatic Colonization. Cell. Rep..

[76] Reichl P., Dengler M., van Zijl F., Huber H., Fuhrlinger G., Reichel C., Sieghart W., Peck-Radosavljevic M., Grubinger M., Mikulits W. (2015). Axl activates autocrine transforming growth factor-β signaling in hepatocellular carcinoma. Hepatology.

[77] Gupta P., Srivastava S.K. (2014). HER2 mediated de novo production of TGF-β leads to SNAIL driven epithelial-to-mesenchymal transition and metastasis of breast cancer. Mol. Oncol..

[78] O’Beirne S.L., Walsh S.M., Fabre A., Reviriego C., Worrell J.C., Counihan I.P., Lumsden R.V., Cramton-Barnes J., Belperio J.A., Donnelly S.C. (2015). CXCL9 Regulates TGF-β1-Induced Epithelial to Mesenchymal Transition in Human Alveolar Epithelial Cells. J. Immunol..

[79] Tian H., Liu J., Chen J., Gatza M.L., Blobe G.C. (2015). Fibulin-3 is a novel TGF-β pathway inhibitor in the breast cancer microenvironment. Oncogene.

[80] Doi S., Zou Y., Togao O., Pastor J.V., John G.B., Wang L., Shiizaki K., Gotschall R., Schiavi S., Yorioka N. (2011). Klotho inhibits transforming growth factor-β1 (TGF-β1) signaling and suppresses renal fibrosis and cancer metastasis in mice. J. Biol. Chem..

[81] Wu Y.Y., Peck K., Chang Y.L., Pan S.H., Cheng Y.F., Lin J.C., Yang R.B., Hong T.M., Yang P.C. (2011). SCUBE3 is an endogenous TGF-β receptor ligand and regulates the epithelial-mesenchymal transition in lung cancer. Oncogene.

[82] Lin Y.C., Lee Y.C., Li L.H., Cheng C.J., Yang R.B. (2014). Tumor suppressor SCUBE2 inhibits breast-cancer cell migration and invasion through the reversal of epithelial-mesenchymal transition. J. Cell. Sci..

[83] Sabbah M., Prunier C., Ferrand N., Megalophonos V., Lambein K., De Wever O., Nazaret N., Lachuer J., Dumont S., Redeuilh G. (2011). CCN5, a novel transcriptional repressor of the transforming growth factor β signaling pathway. Mol. Cell. Biol..

[84] Micalizzi D.S., Christensen K.L., Jedlicka P., Coletta R.D., Baron A.E., Harrell J.C., Horwitz K.B., Billheimer D., Heichman K.A., Welm A.L. (2009). The Six1 homeoprotein induces human mammary carcinoma cells to undergo epithelial-mesenchymal transition and metastasis in mice through increasing TGF-β signaling. J. Clin. Investig..

[85] Meyer A.E., Gatza C.E., How T., Starr M., Nixon A.B., Blobe G.C. (2014). Role of TGF-β receptor III localization in polarity and breast cancer progression. Mol. Biol. Cell..

[86] Kim Y., Kugler M.C., Wei Y., Kim K.K., Li X., Brumwell A.N., Chapman H.A. (2009). Integrin α3 β1-dependent β-catenin phosphorylation links epithelial Smad signaling to cell contacts. J. Cell. Biol..

[87] Xu Q., Isaji T., Lu Y., Gu W., Kondo M., Fukuda T., Du Y., Gu J. (2012). Roles of *N*-acetylglucosaminyltransferase III in epithelial-to-mesenchymal transition induced by transforming growth factor β1 (TGF-β1) in epithelial cell lines. J. Biol. Chem..

[88] Muthusamy B.P., Budi E.H., Katsuno Y., Lee M.K., Smith S.M., Mirza A.M., Akhurst R.J., Derynck R. (2015). ShcA Protects against Epithelial-Mesenchymal Transition through Compartmentalized Inhibition of TGF-β-Induced Smad Activation. PLoS Biol..

[89] Hwangbo C., Tae N., Lee S., Kim O., Park O.K., Kim J., Kwon S.H., Lee J.H. (2016). Syntenin regulates TGF-β1-induced Smad activation and the epithelial-to-mesenchymal transition by inhibiting caveolin-mediated TGF-β type I receptor internalization. Oncogene.

[90] Zhao L., Lin L., Pan C., Shi M., Liao Y., Bin J., Liao W. (2015). Flotillin-2 promotes nasopharyngeal carcinoma metastasis and is necessary for the epithelial-mesenchymal transition induced by transforming growth factor-β. Oncotarget.

[91] Hoot K.E., Lighthall J., Han G., Lu S.L., Li A., Ju W., Kulesz-Martin M., Bottinger E., Wang X.J. (2008). Keratinocyte-specific Smad2 ablation results in increased epithelial-mesenchymal transition during skin cancer formation and progression. J. Clin. Investig..

[92] Velden J.L., Alcorn J.F., Guala A.S., Badura E.C., Janssen-Heininger Y.M. (2011). c-Jun *N*-terminal kinase 1 promotes transforming growth factor-β1-induced epithelial-to-mesenchymal transition via control of linker phosphorylation and transcriptional activity of Smad3. AM. J. Respir. Cell. Mol. Biol..

[93] Bae E., Kim S.J., Hong S., Liu F., Ooshima A. (2012). Smad3 linker phosphorylation attenuates Smad3 transcriptional activity and TGF-β1/Smad3-induced epithelial-mesenchymal transition in renal epithelial cells. Biochem. Biophys. Res. Commun..

[94] Alcorn J.F., Guala A.S., van der Velden J., McElhinney B., Irvin C.G., Davis R.J., Janssen-Heininger Y.M. (2008). Jun *N*-terminal kinase 1 regulates epithelial-to-mesenchymal transition induced by TGF-β1. J. Cell. Sci..

[95] Yoshida K., Murata M., Yamaguchi T., Matsuzaki K., Okazaki K. (2016). Reversible Human TGF-β Signal Shifting between Tumor Suppression and Fibro-Carcinogenesis: Implications of Smad Phospho-Isoforms for Hepatic Epithelial-Mesenchymal Transitions. J. Clin. Med..

[96] Runyan C.E., Hayashida T., Hubchak S., Curley J.F., Schnaper H.W. (2009). Role of SARA (SMAD anchor for receptor activation) in maintenance of epithelial cell phenotype. J. Biol. Chem..

[97] Chandhoke A.S., Karve K., Dadakhujaev S., Netherton S., Deng L., Bonni S. (2016). The ubiquitin ligase Smurf2 suppresses TGFβ-induced epithelial-mesenchymal transition in a sumoylation-regulated manner. Cell. Death Differ..

[98] Netherton S.J., Bonni S. (2010). Suppression of TGFβ-induced epithelial-mesenchymal transition like phenotype by a PIAS1 regulated sumoylation pathway in NMuMG epithelial cells. PLoS ONE.

[99] Hesling C., Fattet L., Teyre G., Jury D., Gonzalo P., Lopez J., Vanbelle C., Morel A.P., Gillet G., Mikaelian I., Rimokh R. (2011). Antagonistic regulation of EMT by TIF1γ and Smad4 in mammary epithelial cells. EMBO Rep..

[100] Chen N., Balasenthil S., Reuther J., Frayna A., Wang Y., Chandler D.S., Abruzzo L.V., Rashid A., Rodriguez J., Lozano G. (2013). DEAR1 is a chromosome 1p35 tumor suppressor and master regulator of TGF-β-driven epithelial-mesenchymal transition. Cancer Discov..

[101] Shan B., Yao T.P., Nguyen H.T., Zhuo Y., Levy D.R., Klingsberg R.C., Tao H., Palmer M.L., Holder K.N., Lasky J.A. (2008). Requirement of HDAC6 for transforming growth factor-β1-induced epithelial-mesenchymal transition. J. Biol. Chem..

[102] Gu S., Liu Y., Zhu B., Ding K., Yao T.P., Chen F., Zhan L., Xu P., Ehrlich M., Liang T., Lin X., Feng X.-H. (2016). Loss of α-Tubulin Acetylation Is Associated with TGF-β-induced Epithelial-Mesenchymal Transition. J. Biol. Chem..

[103] Yamashita M., Fatyol K., Jin C., Wang X., Liu Z., Zhang Y.E. (2008). TRAF6 mediates Smad-independent activation of JNK and p38 by TGF-β. Mol. Cell.

[104] Strippoli R., Benedicto I., Perez Lozano M.L., Pellinen T., Sandoval P., Lopez-Cabrera M., del Pozo M.A. (2012). Inhibition of transforming growth factor-activated kinase 1 (TAK1) blocks and reverses epithelial to mesenchymal transition of mesothelial cells. PLoS ONE.

[105] Thakur N., Gudey S.K., Marcusson A., Fu J.Y., Bergh A., Heldin C.-H., Landström M. (2014). TGFβ-induced invasion of prostate cancer cells is promoted by c-Jun-dependent transcriptional activation of Snail1. Cell. Cycle.

[106] Lam C.R., Tan C., Teo Z., Tay C.Y., Phua T., Wu Y.L., Cai P.Q., Tan L.P., Chen X., Zhu P., Tan N.S. (2013). Loss of TAK1 increases cell traction force in a ROS-dependent manner to drive epithelial-mesenchymal transition of cancer cells. Cell. Death Dis..

[107] Sundar R., Gudey S.K., Heldin C.-H., Landström M. (2015). TRAF6 promotes TGFβ-induced invasion and cell-cycle regulation via Lys63-linked polyubiquitination of Lys178 in TGF-β type I receptor. Cell. Cycle.

[108] Lamouille S., Connolly E., Smyth J.W., Akhurst R.J., Derynck R. (2012). TGF-β-induced activation of mTOR complex 2 drives epithelial-mesenchymal transition and cell invasion. J. Cell. Sci..

[109] Thien A., Prentzell M.T., Holzwarth B., Klasener K., Kuper I., Boehlke C., Sonntag A.G., Ruf S., Maerz L., Nitschke R. (2015). TSC1 activates TGF-β-Smad2/3 signaling in growth arrest and epithelial-to-mesenchymal transition. Dev. Cell..

[110] Serrano I., McDonald P.C., Lock F.E., Dedhar S. (2013). Role of the integrin-linked kinase (ILK)/Rictor complex in TGFβ-1-induced epithelial-mesenchymal transition (EMT). Oncogene.

[111] Gunaratne A., Thai B.L., Di Guglielmo G.M. (2013). Atypical protein kinase C phosphorylates Par6 and facilitates transforming growth factor β-induced epithelial-to-mesenchymal transition. Mol. Cell. Biol..

[112] Wendt M.K., Schiemann B.J., Parvani J.G., Lee Y.H., Kang Y., Schiemann W.P. (2013). TGF-β stimulates Pyk2 expression as part of an epithelial-mesenchymal transition program required for metastatic outgrowth of breast cancer. Oncogene.

[113] Liang Q., Li L., Zhang J., Lei Y., Wang L., Liu D.X., Feng J., Hou P., Yao R., Zhang Y., Huang B., Lu J. (2013). CDK5 is essential for TGF-β1-induced epithelial-mesenchymal transition and breast cancer progression. Sci. Rep..

[114] Li S., Wang L., Zhao Q., Liu Y., He L., Xu Q., Sun X., Teng L., Cheng H., Ke Y. (2014). SHP2 positively regulates TGF-β1-induced epithelial-mesenchymal transition modulated by its novel interacting protein Hook1. J. Biol. Chem..

[115] Buonato J.M., Lan I.S., Lazzara M.J. (2015). EGF augments TGF-β-induced epithelial-mesenchymal transition by promoting SHP2 binding to GAB1. J. Cell. Sci..

[116] Aoyama D., Hashimoto N., Sakamoto K., Kohnoh T., Kusunose M., Kimura M., Ogata R., Imaizumi K., Kawabe T., Hasegawa Y. (2013). Involvement of TGFβ-induced phosphorylation of the PTEN *C*-terminus on TGFβ-induced acquisition of malignant phenotypes in lung cancer cells. PLoS ONE.

[117] Jiang Y., Feng X., Zheng L., Li S.L., Ge X.Y., Zhang J.G. (2015). Thioredoxin 1 mediates TGF-β-induced epithelial-mesenchymal transition in salivary adenoid cystic carcinoma. Oncotarget.

[118] Shiota M., Zardan A., Takeuchi A., Kumano M., Beraldi E., Naito S., Zoubeidi A., Gleave M.E. (2012). Clusterin mediates TGF-β-induced epithelial-mesenchymal transition and metastasis via Twist1 in prostate cancer cells. Cancer Res..

[119] D'Souza R.C., Knittle A.M., Nagaraj N., van Dinther M., Choudhary C., ten Dijke P., Mann M., Sharma K. (2014). Time-resolved dissection of early phosphoproteome and ensuing proteome changes in response to TGF-β. Sci Signal..

[120] Okayama A., Miyagi Y., Oshita F., Ito H., Nakayama H., Nishi M., Kurata Y., Kimura Y., Ryo A., Hirano H. (2015). Identification of Tyrosine-Phosphorylated Proteins Upregulated during Epithelial-Mesenchymal Transition Induced with TGF-β. J. Proteome Res..

[121] Zadran S., Arumugam R., Herschman H., Phelps M.E., Levine R.D. (2014). Surprisal analysis characterizes the free energy time course of cancer cells undergoing epithelial-to-mesenchymal transition. Proc. Natl. Acad. Sci. USA.

[122] Jiang L., Xiao L., Sugiura H., Huang X., Ali A., Kuro-o M., Deberardinis R.J., Boothman D.A. (2015). Metabolic reprogramming during TGFβ1-induced epithelial-to-mesenchymal transition. Oncogene.

[123] Burk U., Schubert J., Wellner U., Schmalhofer O., Vincan E., Spaderna S., Brabletz T. (2008). A reciprocal repression between ZEB1 and members of the miR-200 family promotes EMT and invasion in cancer cells. EMBO Rep..

[124] Gregory P.A., Bert A.G., Paterson E.L., Barry S.C., Tsykin A., Farshid G., Vadas M.A., Khew-Goodall Y., Goodall G.J. (2008). The miR-200 family and miR-205 regulate epithelial to mesenchymal transition by targeting ZEB1 and SIP1. Nat. Cell. Biol..

[125] Korpal M., Lee E.S., Hu G., Kang Y. (2008). The miR-200 family inhibits epithelial-mesenchymal transition and cancer cell migration by direct targeting of E-cadherin transcriptional repressors ZEB1 and ZEB2. J. Biol. Chem..

[126] Gregory P.A., Bracken C.P., Smith E., Bert A.G., Wright J.A., Roslan S., Morris M., Wyatt L., Farshid G., Lim Y.Y. (2011). An autocrine TGF-β/ZEB/miR-200 signaling network regulates establishment and maintenance of epithelial-mesenchymal transition. Mol. Biol. Cell..

[127] Gibbons D.L., Lin W., Creighton C.J., Rizvi Z.H., Gregory P.A., Goodall G.J., Thilaganathan N., Du L., Zhang Y., Pertsemlidis A. (2009). Contextual extracellular cues promote tumor cell EMT and metastasis by regulating miR-200 family expression. Genes Dev..

[128] Zhang J., Tian X.J., Zhang H., Teng Y., Li R., Bai F., Elankumaran S., Xing J. (2014). TGF-β-induced epithelial-to-mesenchymal transition proceeds through stepwise activation of multiple feedback loops. Sci. Signal..

[129] Chen Y., Xiao Y., Ge W., Zhou K., Wen J., Yan W., Wang Y., Wang B., Qu C., Wu J., Xu L., Cai W. (2013). miR-200b inhibits TGF-β1-induced epithelial-mesenchymal transition and promotes growth of intestinal epithelial cells. Cell. Death Dis..

[130] Ding X., Park S.I., McCauley L.K., Wang C.Y. (2013). Signaling between transforming growth factor β (TGF-β) and transcription factor SNAI2 represses expression of microRNA miR-203 to promote epithelial-mesenchymal transition and tumor metastasis. J. Biol. Chem..

[131] Iliopoulos D., Polytarchou C., Hatziapostolou M., Kottakis F., Maroulakou I.G., Struhl K., Tsichlis P.N. (2009). MicroRNAs differentially regulated by Akt isoforms control EMT and stem cell renewal in cancer cells. Sci. Signal..

[132] Eades G., Yao Y., Yang M., Zhang Y., Chumsri S., Zhou Q. (2011). miR-200a regulates SIRT1 expression and epithelial to mesenchymal transition (EMT)-like transformation in mammary epithelial cells. J. Biol. Chem..

[133] Simic P., Williams E.O., Bell E.L., Gong J.J., Bonkowski M., Guarente L. (2013). SIRT1 suppresses the epithelial-to-mesenchymal transition in cancer metastasis and organ fibrosis. Cell. Rep..

[134] Kong D., Li Y., Wang Z., Banerjee S., Ahmad A., Kim H.R., Sarkar F.H. (2009). miR-200 regulates PDGF-D-mediated epithelial-mesenchymal transition, adhesion, and invasion of prostate cancer cells. Stem Cells.

[135] Turcatel G., Rubin N., El-Hashash A., Warburton D. (2012). MIR-99a and MIR-99b modulate TGF-β induced epithelial to mesenchymal plasticity in normal murine mammary gland cells. PLoS ONE.

[136] Zhang F., Luo Y., Shao Z., Xu L., Liu X., Niu Y., Shi J., Sun X., Liu Y., Ding Y., Zhao L. (2016). MicroRNA-187, a downstream effector of TGFβ pathway, suppresses Smad-mediated epithelial-mesenchymal transition in colorectal cancer. Cancer Lett..

[137] Subramanyam D., Lamouille S., Judson R.L., Liu J.Y., Bucay N., Derynck R., Blelloch R. (2011). Multiple targets of miR-302 and miR-372 promote reprogramming of human fibroblasts to induced pluripotent stem cells. Nat. Biotechnol..

[138] Lipchina I., Elkabetz Y., Hafner M., Sheridan R., Mihailovic A., Tuschl T., Sander C., Studer L., Betel D. (2011). Genome-wide identification of microRNA targets in human ES cells reveals a role for miR-302 in modulating BMP response. Genes Dev..

[139] Liao B., Bao X., Liu L., Feng S., Zovoilis A., Liu W., Xue Y., Cai J., Guo X., Qin B. (2011). MicroRNA cluster 302–367 enhances somatic cell reprogramming by accelerating a mesenchymal-to-epithelial transition. J. Biol. Chem..

[140] Johansson J., Berg T., Kurzejamska E., Pang M.F., Tabor V., Jansson M., Roswall P., Pietras K., Sund M., Religa P. (2013). MiR-155-mediated loss of C/EBPβ shifts the TGF-β response from growth inhibition to epithelial-mesenchymal transition, invasion and metastasis in breast cancer. Oncogene.

[141] Richards E.J., Zhang G., Li Z.P., Permuth-Wey J., Challa S., Li Y., Kong W., Dan S., Bui M.M., Coppola D. (2015). Long non-coding RNAs (LncRNA) regulated by transforming growth factor (TGF) β: LncRNA-hit-mediated TGFβ-induced epithelial to mesenchymal transition in mammary epithelia. J. Biol. Chem..

[142] Fan Y., Shen B., Tan M., Mu X., Qin Y., Zhang F., Liu Y. (2014). TGF-β-Induced Upregulation of malat1 Promotes Bladder Cancer Metastasis by Associating with suz12. Clin. Cancer Res..

[143] Yuan J.H., Yang F., Wang F., Ma J.Z., Guo Y.J., Tao Q.F., Liu F., Pan W., Wang T.T., Zhou C.C. (2014). A long noncoding RNA activated by TGF-β promotes the invasion-metastasis cascade in hepatocellular carcinoma. Cancer Cell..

[144] Tay Y., Rinn J., Pandolfi P.P. (2014). The multilayered complexity of ceRNA crosstalk and competition. Nature.

[145] Chaudhury A., Hussey G.S., Ray P.S., Jin G., Fox P.L., Howe P.H. (2010). TGF-β-mediated phosphorylation of hnRNP E1 induces EMT via transcript-selective translational induction of Dab2 and ILEI. Nat. Cell. Biol..

[146] Hussey G.S., Chaudhury A., Dawson A.E., Lindner D.J., Knudsen C.R., Wilce M.C., Merrick W.C., Howe P.H. (2011). Identification of an mRNP complex regulating tumorigenesis at the translational elongation step. Mol. Cell.

[147] Howley B.V., Hussey G.S., Link L.A., Howe P.H. (2016). Translational regulation of inhibin betaA by TGFβ via the RNA-binding protein hnRNP E1 enhances the invasiveness of epithelial-to-mesenchymal transitioned cells. Oncogene.

[148] Nasarre P., Gemmill R.M., Potiron V.A., Roche J., Lu X., Baron A.E., Korch C., Garrett-Mayer E., Lagana A., Howe P.H. (2013). Neuropilin-2 is upregulated in lung cancer cells during TGF-β1-induced epithelial-mesenchymal transition. Cancer Res..

[149] Wittmann P., Grubinger M., Groger C., Huber H., Sieghart W., Peck-Radosavljevic M., Mikulits W. (2015). Neuropilin-2 induced by transforming growth factor-β augments migration of hepatocellular carcinoma cells. BMC Cancer.

[150] Hussey G.S., Link L.A., Brown A.S., Howley B.V., Chaudhury A., Howe P.H. (2012). Establishment of a TGFβ-induced post-transcriptional EMT gene signature. PLoS One.

[151] Dave N., Guaita-Esteruelas S., Gutarra S., Frias A., Beltran M., Peiro S., de Herreros A.G. (2011). Functional cooperation between Snail1 and twist in the regulation of ZEB1 expression during epithelial to mesenchymal transition. J. Biol. Chem..

[152] Medici D., Hay E.D., Olsen B.R. (2008). Snail and Slug promote epithelial-mesenchymal transition through β-catenin-T-cell factor-4-dependent expression of transforming growth factor-β3. Mol. Biol. Cell..

[153] Taube J.H., Herschkowitz J.I., Komurov K., Zhou A.Y., Gupta S., Yang J., Hartwell K., Onder T.T., Gupta P.B., Evans K.W. (2010). Core epithelial-to-mesenchymal transition interactome gene-expression signature is associated with claudin-low and metaplastic breast cancer subtypes. Proc. Natl. Acad. Sci. USA.

[154] Thuault S., Tan E.-J., Peinado H., Cano A., Heldin C.-H., Moustakas A. (2008). HMGA2 and Smads co-regulate SNAIL1 expression during induction of epithelial-to-mesenchymal transition. J. Biol. Chem..

[155] Chang H., Liu Y., Xue M., Liu H., Du S., Zhang L., Wang P. (2016). Synergistic action of master transcription factors controls epithelial-to-mesenchymal transition. Nucleic Acids Res..

[156] Tan E.-J., Thuault S., Caja L., Carletti T., Heldin C.-H., Moustakas A. (2012). Regulation of transcription factor Twist expression by the DNA architectural protein high mobility group A2 during epithelial-to-mesenchymal transition. J. Biol. Chem..

[157] Haraguchi M., Sato M., Ozawa M. (2015). CRISPR/Cas9n-Mediated Deletion of the Snail 1Gene (*SNAI1*) Reveals Its Role in Regulating Cell Morphology, Cell-Cell Interactions, and Gene Expression in Ovarian Cancer (RMG-1) Cells. PLoS ONE.

[158] Li Y., Zhao Z., Xu C., Zhou Z., Zhu Z., You T. (2014). HMGA2 induces transcription factor Slug expression to promote epithelial-to-mesenchymal transition and contributes to colon cancer progression. Cancer Lett..

[159] Vincent T., Neve E.P.A., Johnson J.R., Kukalev A., Rojo F., Albanell J., Pietras K., Virtanen I., Philipson L., Leopold P.L. (2009). A SNAIL1-SMAD3/4 transcriptional repressor complex promotes TGF-β mediated epithelial-mesenchymal transition. Nat. Cell. Biol..

[160] Tan E.J., Kahata K., Idås O., Thuault S., Heldin C.-H., Moustakas A. (2015). The high mobility group A2 protein epigenetically silences the Cdh1 gene during epithelial-to-mesenchymal transition. Nucleic Acids Res..

[161] Berx G., Raspe E., Christofori G., Thiery J.P., Sleeman J.P. (2007). Pre-EMTing metastasis? Recapitulation of morphogenetic processes in cancer. Clin. Exp. Metastasis.

[162] Shi Z., Li X., Wu D., Tang R., Chen R., Xue S., Sun X. (2015). Silencing of HMGA2 suppresses cellular proliferation, migration, invasion, and epithelial-mesenchymal transition in bladder cancer. Tumour Biol..

[163] Thuault S., Valcourt U., Petersen M., Manfioletti G., Heldin C.-H., Moustakas A. (2006). Transforming growth factor-β employs HMGA2 to elicit epithelial-mesenchymal transition. J. Cell. Biol..

[164] Xia Y.Y., Yin L., Jiang N., Guo W.J., Tian H., Jiang X.S., Wu J., Chen M., Wu J.Z., He X. (2015). Downregulating HMGA2 attenuates epithelial-mesenchymal transition-induced invasion and migration in nasopharyngeal cancer cells. Biochem. Biophys. Res. Commun..

[165] Morishita A., Zaidi M.R., Mitoro A., Sankarasharma D., Szabolcs M., Okada Y., D’Armiento J., Chada K. (2013). HMGA2 is a driver of tumor metastasis. Cancer Res..

[166] Dhasarathy A., Phadke D., Mav D., Shah R.R., Wade P.A. (2011). The transcription factors snail and slug activate the transforming growth factor-β signaling pathway in breast cancer. PLoS ONE.

[167] Zhou B., Liu Y., Kahn M., Ann D.K., Han A., Wang H., Nguyen C., Flodby P., Zhong Q., Krishnaveni M.S. (2012). Interactions between β-catenin and transforming growth factor-β signaling pathways mediate epithelial-mesenchymal transition and are dependent on the transcriptional co-activator cAMP-response element-binding protein (CREB)-binding protein (CBP). J. Biol. Chem..

[168] Tian X., Zhang J., Tan T.K., Lyons J.G., Zhao H., Niu B., Lee S.R., Tsatralis T., Zhao Y., Wang Y. (2013). Association of β-catenin with P-Smad3 but not LEF-1 dissociates in vitro profibrotic from anti-inflammatory effects of TGF-β1. J. Cell. Sci..

[169] Gal A., Sjöblom T., Fedorova L., Imreh S., Beug H., Moustakas A. (2008). Sustained TGFβ exposure suppresses Smad and non-Smad signalling in mammary epithelial cells, leading to EMT and inhibition of growth arrest and apoptosis. Oncogene.

[170] Liu X., Sun H., Qi J., Wang L., He S., Liu J., Feng C., Chen C., Li W., Guo Y. (2013). Sequential introduction of reprogramming factors reveals a time-sensitive requirement for individual factors and a sequential EMT-MET mechanism for optimal reprogramming. Nat. Cell. Biol..

[171] Smith A.P., Verrecchia A., Faga G., Doni M., Perna D., Martinato F., Guccione E., Amati B. (2009). A positive role for Myc in TGFβ-induced Snail transcription and epithelial-to-mesenchymal transition. Oncogene.

[172] Liu Y.N., Abou-Kheir W., Yin J.J., Fang L., Hynes P., Casey O., Hu D., Wan Y., Seng V., Sheppard-Tillman H. (2012). Critical and reciprocal regulation of KLF4 and SLUG in transforming growth factor β-initiated prostate cancer epithelial-mesenchymal transition. Mol. Cell. Biol..

[173] Ali A., Zhang P., Liangfang Y., Wenshe S., Wang H., Lin X., Dai Y., Feng X.H., Moses R., Wang D. (2015). KLF17 empowers TGF-β/Smad signaling by targeting Smad3-dependent pathway to suppress tumor growth and metastasis during cancer progression. Cell. Death Dis..

[174] Saito R.A., Watabe T., Horiguchi K., Kohyama T., Saitoh M., Nagase T., Miyazono K. (2009). Thyroid transcription factor-1 inhibits transforming growth factor-β-mediated epithelial-to-mesenchymal transition in lung adenocarcinoma cells. Cancer Res..

[175] Balli D., Ustiyan V., Zhang Y., Wang I.C., Masino A.J., Ren X., Whitsett J.A., Kalinichenko V.V., Kalin T.V. (2013). Foxm1 transcription factor is required for lung fibrosis and epithelial-to-mesenchymal transition. EMBO J..

[176] Zheng X., Vittar N.B., Gai X., Fernandez-Barrena M.G., Moser C.D., Hu C., Almada L.L., McCleary-Wheeler A.L., Elsawa S.F., Vrabel A.M. (2012). The transcription factor GLI1 mediates TGFβ1 driven EMT in hepatocellular carcinoma via a SNAI1-dependent mechanism. PLoS ONE.

[177] Saitoh M., Endo K., Furuya S., Minami M., Fukasawa A., Imamura T., Miyazawa K. (2016). STAT3 integrates cooperative Ras and TGF-β signals that induce Snail expression. Oncogene.

[178] Yin X., Wolford C.C., Chang Y.S., McConoughey S.J., Ramsey S.A., Aderem A., Hai T. (2010). ATF3, an adaptive-response gene, enhances TGFβ signaling and cancer-initiating cell features in breast cancer cells. J. Cell. Sci..

[179] Cho K.H., Jeong K.J., Shin S.C., Kang J., Park C.G., Lee H.Y. (2013). STAT3 mediates TGF-β1-induced TWIST1 expression and prostate cancer invasion. Cancer Lett..

[180] Tsuji T., Ibaragi S., Shima K., Hu M.G., Katsurano M., Sasaki A., Hu G.F. (2008). Epithelial-mesenchymal transition induced by growth suppressor p12^CDK2−AP1^ promotes tumor cell local invasion but suppresses distant colony growth. Cancer Res..

[181] Brandl M., Seidler B., Haller F., Adamski J., Schmid R.M., Saur D., Schneider G. (2010). IKKα controls canonical TGFβ-SMAD signaling to regulate genes expressing SNAIL and SLUG during EMT in panc1 cells. J. Cell. Sci..

[182] Horiguchi K., Shirakihara T., Nakano A., Imamura T., Miyazono K., Saitoh M. (2009). Role of Ras signaling in the induction of snail by transforming growth factor-β. J. Biol. Chem..

[183] Franco D.L., Mainez J., Vega S., Sancho P., Murillo M.M., de Frutos C.A., Del Castillo G., Lopez-Blau C., Fabregat I., Nieto M.A. (2010). Snail1 suppresses TGF-β-induced apoptosis and is sufficient to trigger EMT in hepatocytes. J. Cell. Sci..

[184] David C.J., Huang Y.H., Chen M., Su J., Zou Y., Bardeesy N., Iacobuzio-Donahue C.A., Massagué J. (2016). TGF-β Tumor Suppression through a Lethal EMT. Cell.

[185] Tiwari N., Tiwari V.K., Waldmeier L., Balwierz P.J., Arnold P., Pachkov M., Meyer-Schaller N., Schubeler D., van Nimwegen E., Christofori G. (2013). Sox4 is a master regulator of epithelial-mesenchymal transition by controlling Ezh2 expression and epigenetic reprogramming. Cancer Cell..

[186] Zhang J., Liang Q., Lei Y., Yao M., Li L., Gao X., Feng J., Zhang Y., Gao H., Liu D.X., Lu J., Huang B. (2012). SOX4 induces epithelial-mesenchymal transition and contributes to breast cancer progression. Cancer Res..

[187] Vervoort S.J., Lourenco A.R., van Boxtel R., Coffer P.J. (2013). SOX4 mediates TGF-β-induced expression of mesenchymal markers during mammary cell epithelial to mesenchymal transition. PLoS ONE.

[188] Juang Y.L., Jeng Y.M., Chen C.L., Lien H.C. (2016). PRRX2 as a novel TGF-β-induced factor enhances invasion and migration in mammary epithelial cell and correlates with poor prognosis in breast cancer. Mol. Carcinog..

[189] Cesi V., Casciati A., Sesti F., Tanno B., Calabretta B., Raschella G. (2011). TGFβ-induced c-Myb affects the expression of EMT-associated genes and promotes invasion of ER^+^ breast cancer cells. Cell. Cycle.

[190] Kowanetz M., Valcourt U., Bergström R., Heldin C.-H., Moustakas A. (2004). Id2 and Id3 define the potency of cell proliferation and differentiation responses to transforming growth factor β and bone morphogenetic protein. Mol. Cell. Biol..

[191] Kondo M., Cubillo E., Tobiume K., Shirakihara T., Fukuda N., Suzuki H., Shimizu K., Takehara K., Cano A., Saitoh M. (2004). A role for Id in the regulation of TGF-β-induced epithelial-mesenchymal transdifferentiation. Cell. Death Differ..

[192] Gervasi M., Bianchi-Smiraglia A., Cummings M., Zheng Q., Wang D., Liu S., Bakin A.V. (2012). JunB contributes to Id2 repression and the epithelial-mesenchymal transition in response to transforming growth factor-β. J. Cell. Biol..

[193] Siegel P.M., Shu W., Massagué J. (2003). Mad upregulation and Id2 repression accompany transforming growth factor (TGF)-β-mediated epithelial cell growth suppression. J. Biol. Chem..

[194] Romagnoli M., Belguise K., Yu Z., Wang X., Landesman-Bollag E., Seldin D.C., Chalbos D., Barille-Nion S., Jezequel P., Seldin M.L. (2012). Epithelial-to-mesenchymal transition induced by TGF-β1 is mediated by Blimp-1-dependent repression of BMP-5. Cancer Res..

[195] Stankic M., Pavlovic S., Chin Y., Brogi E., Padua D., Norton L., Massagué J., Benezra R. (2013). TGF-β-Id1 Signaling Opposes Twist1 and Promotes Metastatic Colonization via a Mesenchymal-to-Epithelial Transition. Cell. Rep..

[196] Motizuki M., Saitoh M., Miyazawa K. (2015). Maid is a negative regulator of transforming growth factor-β-induced cell migration. J. Biochem..

[197] Cieply B., Riley P., Pifer P.M., Widmeyer J., Addison J.B., Ivanov A.V., Denvir J., Frisch S.M. (2012). Suppression of the epithelial-mesenchymal transition by Grainyhead-like-2. Cancer Res..

[198] Cieply B., Farris J., Denvir J., Ford H.L., Frisch S.M. (2013). Epithelial-mesenchymal transition and tumor suppression are controlled by a reciprocal feedback loop between ZEB1 and Grainyhead-like-2. Cancer Res..

[199] Watanabe K., Villarreal-Ponce A., Sun P., Salmans M.L., Fallahi M., Andersen B., Dai X. (2014). Mammary morphogenesis and regeneration require the inhibition of EMT at terminal end buds by Ovol2 transcriptional repressor. Dev. Cell..

[200] Papageorgis P., Lambert A.W., Ozturk S., Gao F., Pan H., Manne U., Alekseyev Y.O., Thiagalingam A., Abdolmaleky H.M., Lenburg M., Thiagalingam S. (2010). Smad signaling is required to maintain epigenetic silencing during breast cancer progression. Cancer Res..

[201] Dumont N., Wilson M.B., Crawford Y.G., Reynolds P.A., Sigaroudinia M., Tlsty T.D. (2008). Sustained induction of epithelial to mesenchymal transition activates DNA methylation of genes silenced in basal-like breast cancers. Proc. Natl. Acad. Sci. U S A.

[202] McDonald O.G., Wu H., Timp W., Doi A., Feinberg A.P. (2011). Genome-scale epigenetic reprogramming during epithelial-to-mesenchymal transition. Nat. Struct. Mol. Biol..

[203] Tange S., Oktyabri D., Terashima M., Ishimura A., Suzuki T. (2014). JARID2 is involved in transforming growth factor-β-induced epithelial-mesenchymal transition of lung and colon cancer cell lines. PLoS ONE.

[204] Bu F., Liu X., Li J., Chen S., Tong X., Ma C., Mao H., Pan F., Li X., Chen B. (2015). TGF-β1 induces epigenetic silence of TIP30 to promote tumor metastasis in esophageal carcinoma. Oncotarget.

[205] Tang Y.N., Ding W.Q., Guo X.J., Yuan X.W., Wang D.M., Song J.G. (2015). Epigenetic regulation of Smad2 and Smad3 by profilin-2 promotes lung cancer growth and metastasis. Nat. Commun..

[206] Risolino M., Mandia N., Iavarone F., Dardaei L., Longobardi E., Fernandez S., Talotta F., Bianchi F., Pisati F., Spaggiari L. (2014). Transcription factor PREP1 induces EMT and metastasis by controlling the TGF-β-SMAD3 pathway in non-small cell lung adenocarcinoma. Proc. Natl. Acad. Sci. USA.

[207] Akhurst R.J., Hata A. (2012). Targeting the TGFβ signalling pathway in disease. Nat. Rev. Drug Discov..

[208] Park S.Y., Kim M.J., Park S.A., Kim J.S., Min K.N., Kim D.K., Lim W., Nam J.S., Sheen Y.Y. (2015). Combinatorial TGF-β attenuation with paclitaxel inhibits the epithelial-to-mesenchymal transition and breast cancer stem-like cells. Oncotarget.

[209] Zhou B., Buckley S.T., Patel V., Liu Y., Luo J., Krishnaveni M.S., Ivan M., DeMaio L., Kim K.J., Ehrhardt C. (2012). Troglitazone attenuates TGF-β1-induced EMT in alveolar epithelial cells via a PPARγ-independent mechanism. PLoS ONE.

[210] Xi Y., Tan K., Brumwell A.N., Chen S.C., Kim Y.H., Kim T.J., Wei Y., Chapman H.A. (2014). Inhibition of epithelial-to-mesenchymal transition and pulmonary fibrosis by methacycline. Am. J. Respir. Cell. Mol. Biol..

[211] Guo L., Peng W., Tao J., Lan Z., Hei H., Tian L., Pan W., Wang L., Zhang X. (2016). Hydrogen Sulfide Inhibits Transforming Growth Factor-β1-Induced EMT via Wnt/Catenin Pathway. PLoS ONE.

[212] Wu Y., Fu Y., Zheng L., Lin G., Ma J., Lou J., Zhu H., He Q., Yang B. (2014). Nutlin-3 inhibits epithelial-mesenchymal transition by interfering with canonical transforming growth factor-β1-Smad-Snail/Slug axis. Cancer Lett..

[213] Han D., Wu G., Chang C., Zhu F., Xiao Y., Li Q., Zhang T., Zhang L. (2015). Disulfiram inhibits TGF-β-induced epithelial-mesenchymal transition and stem-like features in breast cancer via ERK/NF-κB/Snail pathway. Oncotarget.

[214] Sakamoto T., Kobayashi S., Yamada D., Nagano H., Tomokuni A., Tomimaru Y., Noda T., Gotoh K., Asaoka T., Wada H. (2016). A Histone Deacetylase Inhibitor Suppresses Epithelial-Mesenchymal Transition and Attenuates Chemoresistance in Biliary Tract Cancer. PLoS ONE.

[215] Smith K.A., Zhou B., Avdulov S., Benyumov A., Peterson M., Liu Y., Okon A., Hergert P., Braziunas J., Wagner C.R. (2015). Transforming Growth Factor-β1 Induced Epithelial Mesenchymal Transition is blocked by a chemical antagonist of translation factor eIF4E. Sci. Rep..

[216] Omori K., Hattori N., Senoo T., Takayama Y., Masuda T., Nakashima T., Iwamoto H., Fujitaka K., Hamada H., Kohno N. (2016). Inhibition of Plasminogen Activator Inhibitor-1 Attenuates Transforming Growth Factor-β-Dependent Epithelial Mesenchymal Transition and Differentiation of Fibroblasts to Myofibroblasts. PLoS ONE.

[217] Fu J., Ke X., Tan S., Liu T., Wang S., Ma J., Lu H. (2016). The natural compound codonolactone attenuates TGF-β1-mediated epithelial-to-mesenchymal transition and motility of breast cancer cells. Oncol. Rep..

[218] Zhang L., Cheng X., Gao Y., Zhang C., Bao J., Guan H., Yu H., Lu R., Xu Q., Sun Y. (2016). Curcumin inhibits metastasis in human papillary thyroid carcinoma BCPAP cells via down-regulation of the TGF- β/Smad2/3 signaling pathway. Exp. Cell. Res..

[219] Wu J., Han J., Hou B., Deng C., Wu H., Shen L. (2016). Sulforaphane inhibits TGF-β-induced epithelial-mesenchymal transition of hepatocellular carcinoma cells via the reactive oxygen species-dependent pathway. Oncol. Rep..

[220] Banno A., Garcia D.A., van Baarsel E.D., Metz P.J., Fisch K., Widjaja C.E., Kim S.H., Lopez J., Chang A.N., Geurink P.P. (2016). Downregulation of 26S proteasome catalytic activity promotes epithelial-mesenchymal transition. Oncotarget.

